# Permanent Oxidative Dipole Engineering in Quinoline COFs for Boosting Exciton Dissociation and Oxygen Activation in H_2_O_2_ Photosynthesis

**DOI:** 10.1002/advs.77745

**Published:** 2026-09-16

**Authors:** Yong‐Quan Wu, Zhi‐Bo Zuo, Wei‐Rong Cui

**Affiliations:** ^1^ College of Chemistry and Materials Gannan Normal University Ganzhou People's Republic of China

**Keywords:** covalent organic frameworks, H_2_O_2_, permanent oxidative dipole, photocatalysis

## Abstract

Despite their robust stability, fully conjugated quinoline‐linked covalent organic frameworks suffer from highly delocalized electron distributions and a homogeneous electrostatic potential, which physically cause high exciton binding energies and poor oxygen activation that severely limit photocatalytic H_2_O_2_ production. To fundamentally disrupt this electronic uniformity, we propose a permanent oxidative dipole (POD) strategy, where site‐selective oxidation of quinoline nitrogen atoms, synthesized via one‐pot [4+2] annulation, precisely embeds N^+–^O^−^ dipole pairs into the NQ‐COF_BD_ framework. These POD sites trigger localized charge polarization, producing a triple synergistic effect: a strengthened built‐in electric field (dipole moment increases from 1.08 to 2.08 D), a lowered exciton dissociation barrier (binding energy drops from 44.7 to 20.3 meV), and accelerated charge carrier transport (surface potential rising by a factor of 1.26). Crucially, the POD sites construct spatially decoupled redox dual‐centers that drastically reduce the kinetic barrier of the ORR rate‐determining step, with the *OOH formation energy lowered by 0.77 eV. Consequently, NQ‐COF_BD_‐O achieves a remarkable H_2_O_2_ production rate of 4,569 µmol g^−1^ h^−1^ under visible light without sacrificial agents (a 1.89‐fold enhancement), with an apparent quantum yield of 6.1% at 460 nm, and this strategy is also validated in another quinoline system with a 1.86‐fold improvement.

## Graphical abstract

Permanent oxidative dipole (POD) engineering overcomes the electronic uniformity of quinoline‐linked COFs. By enhancing the dipole moment to 2.08 D and lowering exciton binding energy to 20.3 meV, it constructs a robust built‐in electric field and spatially decoupled redox centers. This bypasses inherent kinetic bottlenecks, achieving a 4569 µmol g^−^^1^ h^−^^1^ H_2_O_2_ yield and 6.1% AQY without sacrificial agents.

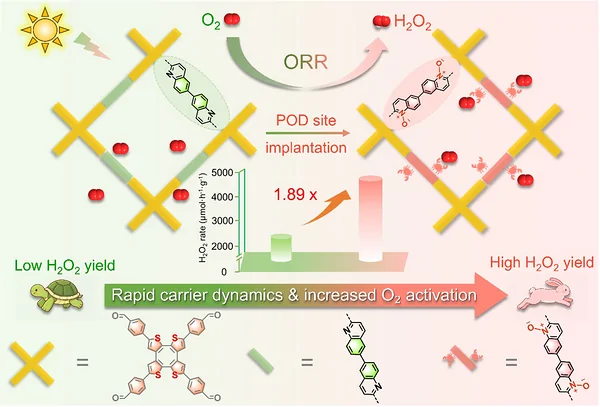

## 1 Introduction

Hydrogen peroxide (H_2_O_2_) is an indispensable commodity chemical for industrial oxidation, environmental remediation, medical disinfection, and energy storage [1, 2, 3]. However, the conventional anthraquinone process suffers from inherent drawbacks, including high energy consumption, complex procedures, reliance on toxic intermediates, and severe byproduct pollution [4, 5, 6]. Solar‐driven photocatalytic H_2_O_2_ production from O_2_ and H_2_O enables green synthesis under ambient conditions and is regarded as the most promising alternative technology [7, 8, 9]. Although various photocatalysts such as graphitic carbon nitride [10, 11] and conjugated microporous polymers [12, 13, 14, 15] have been developed, the H_2_O_2_ production rates of these non‐COF systems under sacrificial‐agent‐free conditions generally remain below 2000 µmol g^−1^ h^−1^. This limited performance originates from the lack of atomic‐level precision in modularly tuning charge separation and surface redox reactions, leaving a substantial gap between current capabilities and industrial requirements. Although some high‐performance COFs have achieved >5000 µmol g^−1^ h^−1^rates in pure water [2, 6], they often lack a universal strategy to simultaneously resolve the inherent exciton dissociation bottleneck and weak oxygen activation of their fully conjugated skeletons.

Covalent organic frameworks (COFs), owing to their atomically precise structural designability, ultrahigh specific surface area, and intrinsic porosity, have emerged as a leading platform for photocatalytic H_2_O_2_ production [16, 17, 18]. Among them, imine‐linked COFs are the most intensively studied because of their mild synthesis conditions and wide monomer availability. Various strategies have been developed to enhance their activity, such as donor–acceptor (D–A) architectures [19, 20, 21], incorporation of photoactive chromophores [22, 23, 24], and metal ion doping [25, 26, 27, 28] to enhance their activity. Nevertheless, the high polarity of the imine bond (C═N) disrupts π‐electron delocalization across the framework, and the resulting discontinuous conjugation causes severe charge recombination [29, 30, 31]. More critically, under the strongly oxidizing environment required for photocatalytic H_2_O_2_ production, the imine bond is susceptible to oxidation to amide linkages, leading to rapid loss of catalytic activity and ambiguous structure–performance relationships [32, 33].

**SCHEME 1:**
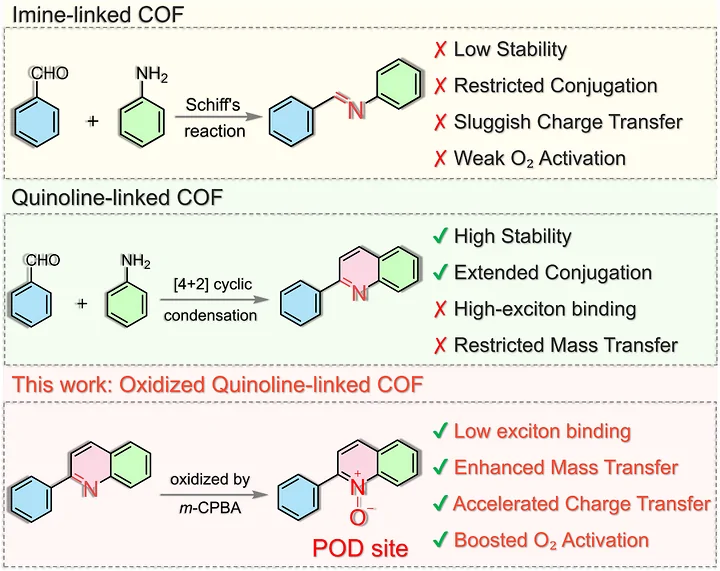
Schematic illustration of the structural evolution and performance comparison of imine‐linked, quinoline‐linked, and the newly developed oxidized quinoline‐linked COFs with pyridine N‐oxide (POD) sites.

To simultaneously address the issues of stability and conjugation, imine‐linked COFs have been converted into quinoline‐linked COFs via intramolecular cyclization assisted by ortho‐positioned groups, yielding fully conjugated frameworks [34, 35]. This transformation represents a generalizable strategy for synergistic enhancement of stability and conjugation. Compared with their imine‐based counterparts, quinoline‐linked COFs exhibit greatly enhanced chemical stability (tolerating 6 M HCl, 6 M NaOH, and 2 M H_2_O_2_) and a significantly extended π‐conjugation, with carrier mobilities increasing by 1–2 orders of magnitude [36, 37]. However, this direct imine‐to‐quinoline conversion, while resolving previous issues, inherits and amplifies new challenges: the fully conjugated rigid planar structure leads to highly delocalized electron distribution and a homogeneous electrostatic surface. This molecular‐level electronic uniformity constitutes the physical origin of two critical bottlenecks—high exciton binding energies that severely hinder the dissociation of photogenerated electron–hole pairs, and the absence of well‐defined electron‐localized active sites that results in intrinsically weak O_2_ adsorption and activation capability. These two critical bottlenecks have so far hindered a major breakthrough in the photocatalytic H_2_O_2_ production performance of quinoline‐linked COFs, as depicted in Scheme 1. Therefore, breaking this homogeneous electronic distribution and precisely constructing local charge gradients within the framework has become the scientific key to achieving a leap in performance.

**SCHEME 2:**
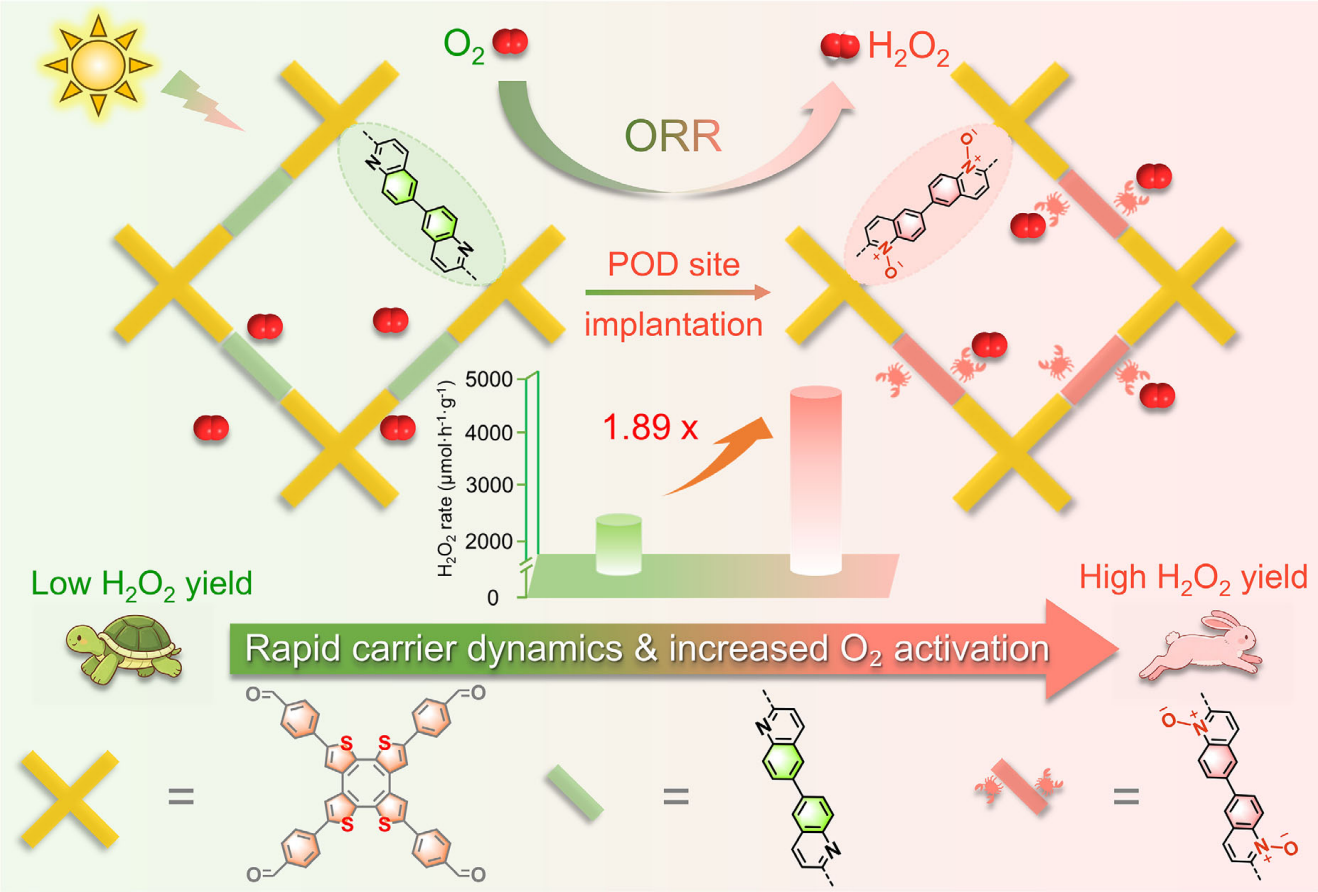
Schematic illustration of the permanent oxidative dipole (POD) strategy implanted in quinoline COFs, illustrating the triple synergistic effects: strengthened built‐in electric field, facilitated exciton dissociation, and enhanced O_2_ activation for efficient photocatalytic H_2_O_2_ production.

Here, we propose and validate the concept of “permanent oxidized dipoles” (POD) to precisely address the aforementioned bottlenecks (Scheme 2). In contrast to conventional random heteroatom doping, our POD strategy achieves site‐selective and non‐destructive structural evolution. A highly crystalline, three‐dimensional, fully conjugated quinoline‐linked COF (NQ‐COF_BD_) was synthesized via a one‐pot, three‐component [4+2] cycloaddition reaction. By selectively bonding an oxygen atom to the N‐terminus of the quinoline, we successfully embedded the POD group into the COF backbone, thereby preparing a quinoline‐N‐oxide COF (NQ‐COF_BD_‐O). The nitrogen cation–oxygen anion pair induces significant local charge polarization, synergistically producing three effects: (i) a substantial reduction in exciton binding energy to promote charge separation, (ii) a significant enhancement of the built‐in electric field to accelerate carrier transport, and (iii) the construction of spatially decoupled redox dual‐centers, in which the terminal oxygen of the N^+^‐O^−^ pair serves as the dedicated active site to improve O_2_ adsorption and surface reaction kinetics. This strategy leads to a 1.89‐fold enhancement in H_2_O_2_ yield (reaching 4569 µmol g^−1^ h^−1^, with an AQY of 6.1% at 460 nm). The feasibility of this POD approach is further validated in a structurally different quinoline COF system (1.86‐fold enhancement), establishing a paradigm for precisely designing permanent dipoles in COFs.

## 2 Results and Discussion

### 2.1 Synthesis and Structural Verification of POD‐Engineered COFs

**FIGURE 1:**
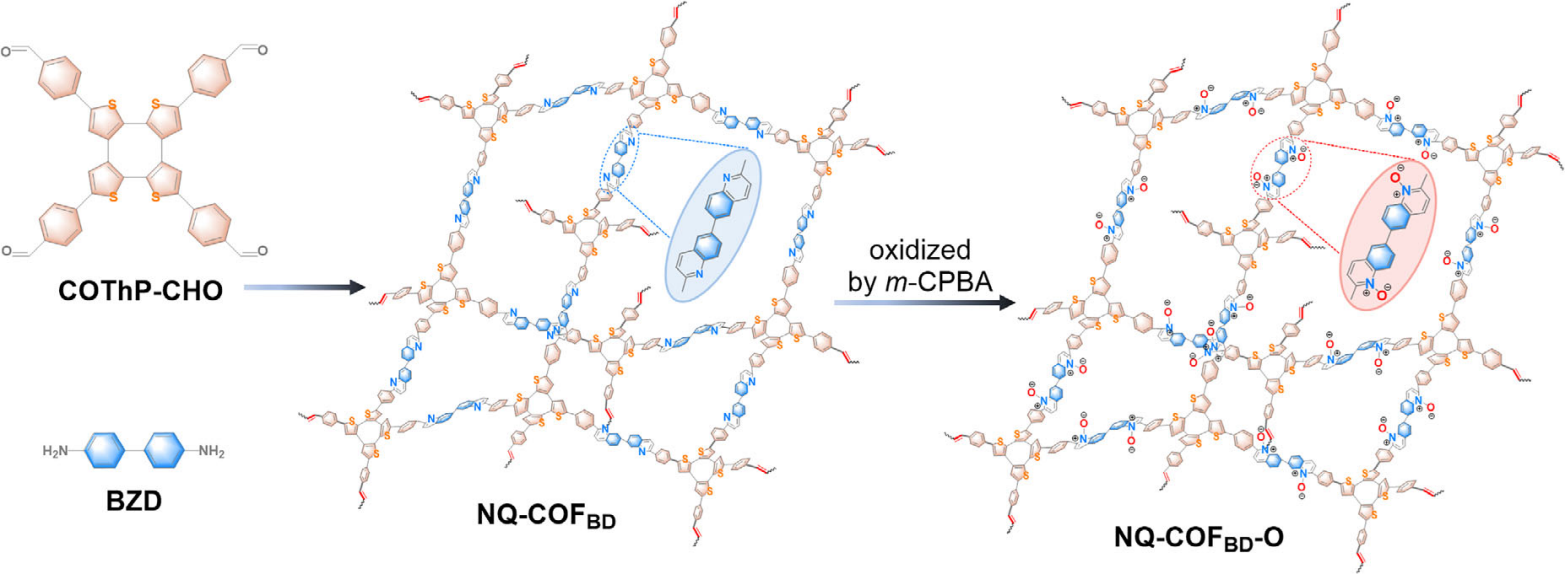
Synthesis and structures of the NQ‐COF_BD_ and NQ‐COF_BD_‐O.

To overcome the core bottlenecks of difficult exciton dissociation and weak oxygen activation in quinoline‐linked COFs, we designed a mild and highly selective post‐oxidation strategy (Figure 1). Using COThP‐CHO and 4,4′‐biphenyldiamine as building blocks, a highly crystalline quinoline‐linked COF (NQ‐COF_BD_) was synthesized via a one‐pot three‐component [4+2] annulation (130°C, 3 days) in 86% yield [38, 39, 40]. Subsequently, site‐selective oxidation of the quinoline nitrogen atoms was performed using *m*‐CPBA at room temperature to afford NQ‐COF_BD_‐O (also 86% yield) [41]. Scanning electron microscopy (SEM) revealed that the oxidation process did not alter the material's microstructure, and the COF framework was fully preserved (Figure S11).

**FIGURE 2:**
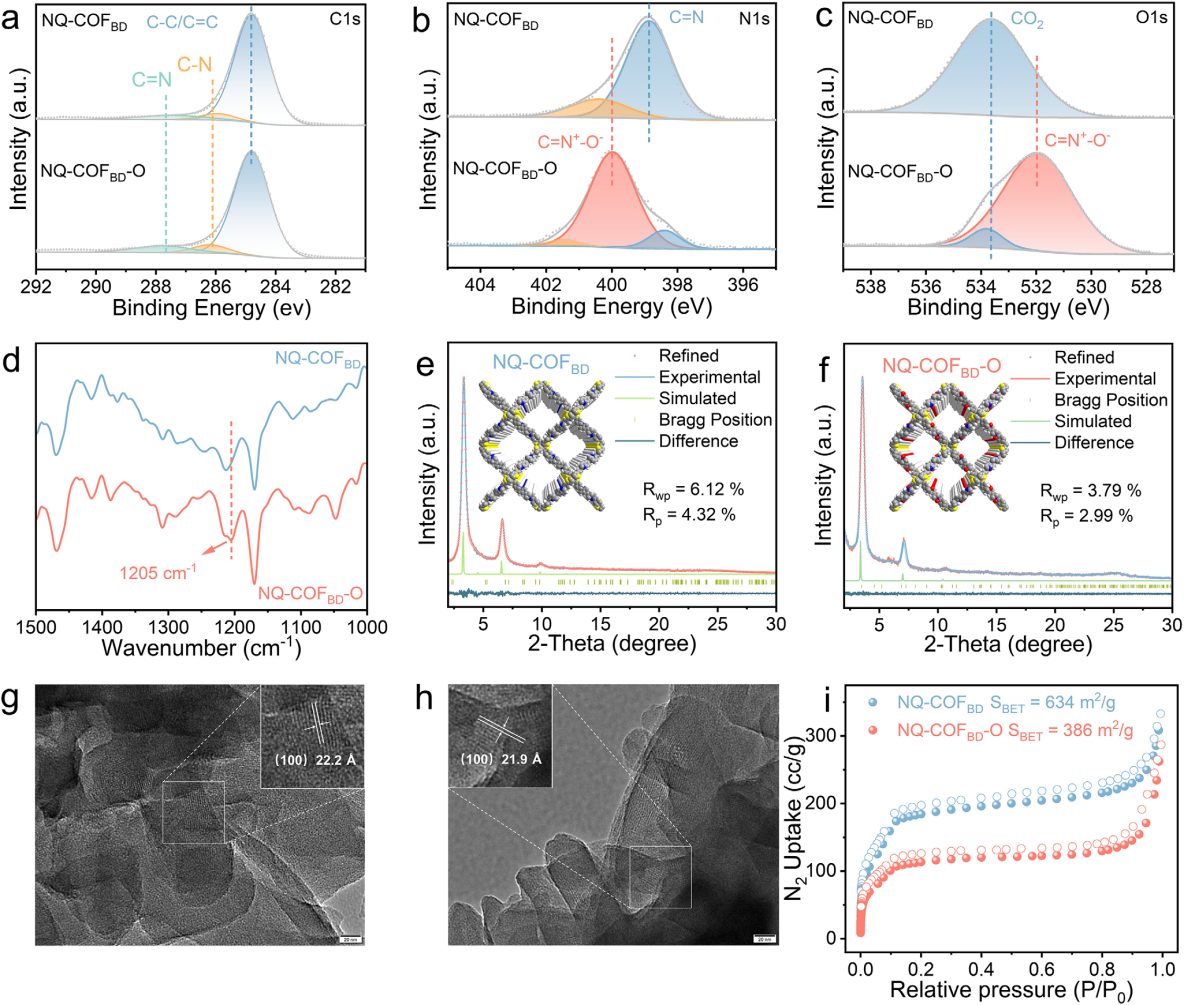
High‐resolution C 1s (a), N 1s (b), and O 1s (c) XPS spectra of NQ‐COF_BD_ and NQ‐COF_BD_‐O. (d) FT‐IR spectra of NQ‐COF_BD_ and NQ‐COF_BD_‐O. Pawley refinements of NQ‐COF_BD_ (e) and NQ‐COF_BD_‐O (f) against their experimental PXRD patterns. HRTEM images of NQ‐COF_BD_ (g) and NQ‐COF_BD_‐O (h) (Inset shows the enlarged view of the selected area). (i) N_2_ adsorption–desorption isotherms of NQ‐COF_BD_ and NQ‐COF_BD_‐O.

The successful introduction of permanent oxidative dipole (POD) sites was confirmed by combined FT‐IR, XPS, and solid‐state ^13^C NMR spectroscopy. XPS analysis shows that, relative to NQ‐COF_BD_, the C 1s peak corresponding to the C═N bonds of the quinoline rings in NQ‐COF_BD_‐O shifts to higher binding energy by 0.2 eV (from 287.6 to 287.8 eV); concurrently, the N 1s peak exhibits a notable positive shift of 1.1 eV (from 398.9 to 400.0 eV), indicative of decreased electron density on nitrogen upon oxygen incorporation. A new O 1s peak appears at 532.0 eV, directly confirming the covalent bonding of oxygen to the quinoline nitrogen atoms (N‐oxide formation) (Figure 2a–c). Quantitative peak fitting of the N 1s spectra reveals that approximately 85.1% of the quinoline nitrogen atoms are converted to the N‐oxide state. Moreover, the S 2p high‐resolution XPS spectra (Figure S12) show that the binding energies of the S 2p_3/2_ and S 2p_1/2_ doublets remain unchanged after oxidation, and no new peaks associated with sulfone or sulfoxide species are observed. Furthermore, XPS survey spectra confirm the absence of residual chlorine (Cl 2p) signals or other carbonaceous impurities, indicating that the *m*‐CPBA oxidant and its byproduct, *m*‐chlorobenzoic acid, were thoroughly removed during the washing process (Figure S13). The successful creation of POD sites was validated by the characteristic N^+^‐O stretching vibration at 1205 cm^−1^ in FT‐IR (Figure 2d) [41]. Solid‐state ^13^C NMR spectroscopy further reveals that the signals assigned to the quinoline C2 and C1 carbons in NQ‐COF_BD_ shift from 145.8 and 156.0 ppm to 143.9 and 154.5 ppm in NQ‐COF_BD_‐O, respectively, confirming the efficient and selective oxidation of the quinoline rings (Figure S14). Elemental analysis and EDS mapping revealed a significant increase in oxygen content after oxidation (Figures S15 and S16 and Table S1). Crucially, Zeta potential measurements revealed a dramatic polarity reversal from +11.7 mV (parent) to −12.2 mV (modified) (Figure S17), confirming the formation of N^+^─O^−^ bonds and the macroscopic pore‐surface charge inversion [42].

Powder X‐ray diffraction (PXRD) combined with structural simulations indicated that both the parent and oxidized materials retained high crystallinity and adopted the same 14‐fold interwoven diamond (dia‐C14) topology (space group PNNN, No. 48) (Figure 2e,f and Table S2). High‐resolution transmission electron microscopy (HRTEM) images show that both samples exhibit distinct lattice fringes at 2.22 and 2.19 nm, respectively, both corresponding to the (011) plane, consistent with the PXRD results (Figure 2g,h).

Nitrogen adsorption–desorption measurements (77 K) showed that both materials exhibited typical type I isotherms, characteristic of microporous structures, while the slight decrease in BET surface area (634 to 386 m^2^ g^−1^) and pore volume (0.51 to 0.44 cm^3^ g^−1^) is typical for post‐synthetic modification (Figures 2i and S18). Thermogravimetric analysis demonstrated that NQ‐COF_BD_‐O possessed excellent thermal stability, with a thermal decomposition temperature as high as 470°C (Figure S19). Chemical stability tests confirmed that both materials exhibited no significant changes in their PXRD and FT‐IR patterns after immersion in highly acidic, alkaline, and oxidizing environments, fully meeting the demands of photocatalytic reactions (Figures S20 and S21).

### 2.2 POD‐Induced Modulation of Photoelectric Properties and Charge Dynamics

**FIGURE 3:**
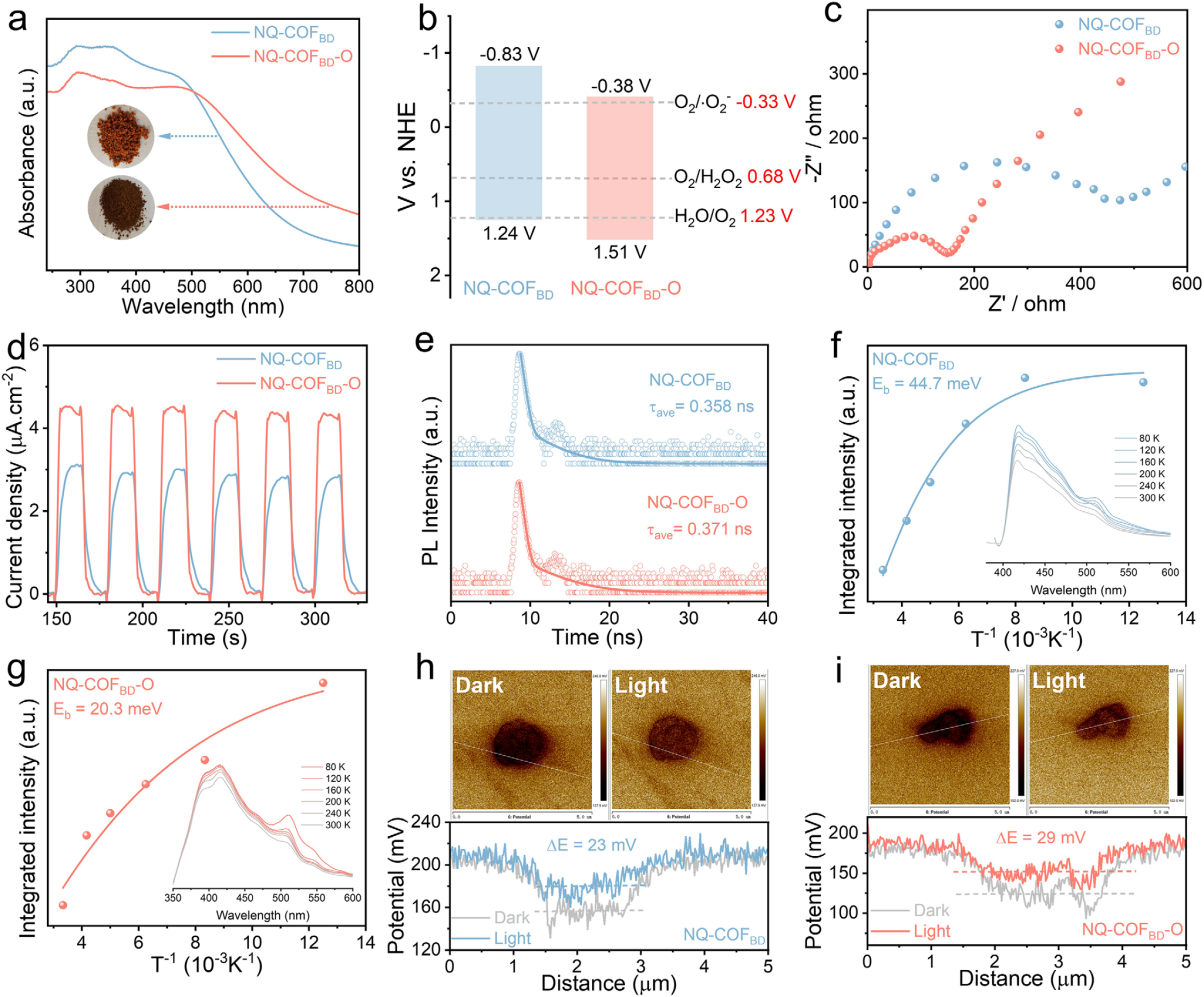
(a) UV–vis DRS, (b) energy band alignments, (c) EIS Nyquist plots, (d) photocurrent responses, (e) TCSPC decay lifetimes; steady‐state PL spectra as a function of reciprocal temperature for (f)NQ‐COF_BD_ and (g)NQ‐COF_BD_‐O (with temperature‐dependent PL insets); KPFM images and corresponding contact potential differences (dark and light) of (h) NQ‐COF_BD_ and (i) NQ‐COF_BD_‐O.

Ultraviolet–visible diffuse reflectance spectroscopy (UV–vis DRS) revealed that the introduction of POD sites significantly narrowed the optical bandgap from 2.07 to 1.89 eV, accompanied by a pronounced red‐shift in absorption (Figures 3a and S22). This enhancement originated from the reconstruction of the framework electron density distribution by the N^+^─O^−^ bonds, which broadened the spectral response range. Mott–Schottky measurements combined with the optical bandgaps determined the band alignment (Figure S23). Both materials were n‐type semiconductors, with the conduction band (CB) and valence band (VB) edges of NQ‐COF_BD_ located at −0.83 and +1.24 V (vs. NHE), respectively. These band positions are thermodynamically capable of simultaneously driving the oxygen reduction reaction (ORR, −0.33 V vs. NHE) and the water oxidation reaction (WOR, +1.23 V vs. NHE), satisfying the requirements for photocatalytic overall H_2_O_2_ synthesis (Figure 3b) [43, 44, 45]. Water contact angle measurements showed that the contact angle of NQ‐COF_BD_‐O was significantly smaller than that of the parent material, consistent with the zeta potential reversal from +11.7 to −12.2 mV. This indicates that the POD sites greatly enhanced surface polarity and hydrophilicity, thereby optimizing the mass transfer kinetics of reactants (·O_2_^−^, H^+^) and products (Figure S24) [42].

We further quantitatively resolved the modulation of charge carrier behavior by the POD sites using electrochemical and spectroscopic techniques. Compared to the parent material, NQ‐COF_BD_‐O exhibited a significantly reduced Nyquist semicircle diameter and a much higher transient photocurrent density, indicating accelerated interfacial charge transfer (Figure 3c,d). Time‐correlated single‐photon counting (TCSPC) and steady‐state photoluminescence (PL) spectroscopy results also indicate that the POD site prolongs carrier lifetime and enhances exciton dissociation efficiency (Figures 3e and S25).

The differences in carrier behavior observed in the above experiments can be further explained by theoretical calculations: we used density functional theory (DFT) to simulate the surface electrostatic potential (ESP) distribution of the material (Figure S26). In NQ‐COF_BD_, the negative charge is distributed across the quinoline ring and adjacent benzene rings, while the positive charge is uniformly dispersed, resulting in weak fluctuations in the electrostatic potential. Upon the introduction of POD sites, the negative charge in NQ‐COF_BD_‐O is highly localized at the terminal oxygen atom of the N^+^─O^−^ bond, and the positive charge is entirely concentrated in the COTh ring units, leading to an extremely asymmetric spatial charge separation. This charge redistribution dramatically increases the molecular dipole moment from 1.08 Debye to 2.08 Debye (a 1.9‐fold enhancement), inducing a strong oriented built‐in electric field within the COF framework that provides a crucial driving force for long‐range transport of photogenerated carriers. Quantitative confirmation was obtained via temperature‐dependent photoluminescence spectroscopy: The exciton binding energy (E_b_) decreased significantly from 44.7 meV in the parent COF to 20.3 meV in NQ‐COF_BD_‐O (a reduction of 54.6%), demonstrating that the POD sites can effectively promote the dissociation of excitons into free charge carriers (Figure 3f,g) [46], effectively overcoming the challenge of difficult exciton dissociation in quinoline‐linked COFs. We further quantitatively characterized the photovoltage on the material surface using Kelvin probe force microscopy (KPFM) (Figure 3h,i), verifying the role of the POD sites in promoting charge transport at the microscopic photoelectric potential level. The ΔE of NQ‐COF_BD_‐O was 29 mV, significantly higher than that of NQ‐COF_BD_ (23 mV); This confirms that the POD sites endow NQ‐COF_BD_‐O with higher charge mobility and rapid charge transfer capability. In summary, the introduction of N^+^─O^−^ polar sites overcomes the charge dynamics bottleneck in quinoline‐linked COFs, laying a solid foundation for the efficient photocatalytic reduction of O_2_ to H_2_O_2_.

### 2.3 Enhanced Photocatalytic H_2_O_2_ Production Enabled by POD Sites

**FIGURE 4:**
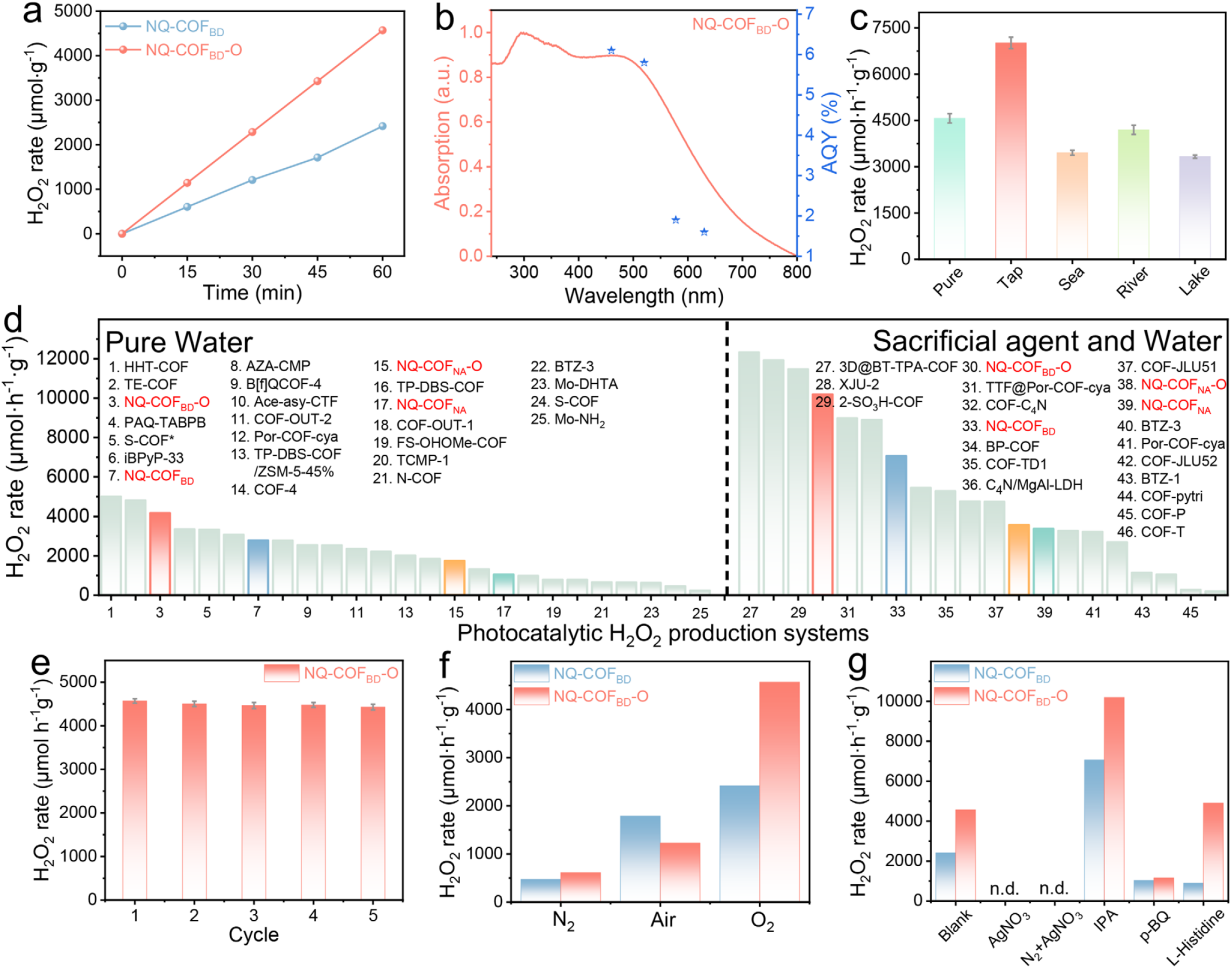
(a) Time‐dependent H_2_O_2_ production over NQ‐COFs under visible‐light (1 h). (b) AQYs of NQ‐COF_BD_‐O at 460, 520, 578, and 630 nm. (c) Photocatalytic H_2_O_2_ production of NQ‐COF_BD_‐O in various water sources under a Xe lamp. (d) Comparison of photocatalytic H_2_O_2_ production with reported COF‐based photocatalysts. (e) Recycling tests for photocatalytic H_2_O_2_ production over NQ‐COF_BD_‐O.(f) Photocatalytic H_2_O_2_ generation over NQ‐COF_BD_ and NQ‐COF_BD_‐O under N_2_, air, and O_2_. (g) Photocatalytic H_2_O_2_ generation over NQ‐COF_BD_ and NQ‐COF_BD_‐O with different sacrificial agents.

To rule out the potential light‐shielding effect and avoid artifacts, we first optimized the catalyst loading by evaluating the solid‐to‐liquid ratios (1:3, 1:5, 1:10, 1:15, and 1:20) for the photocatalytic H_2_O_2_ production (Figures S27 and S28). The optimal performance was achieved at a ratio of 1:15, which was therefore selected for all subsequent photocatalytic evaluations. To verify the actual enhancement effect of the POD site on the photocatalytic H_2_O_2_ production of the quinoline COF, we systematically compared the activities of NQ‐COF_BD_ and NQ‐COF_BD_‐O under visible light irradiation at room temperature (300 W xenon lamp, λ ≥ 420 nm), using O_2_‐saturated water without a sacrificial agent as the reaction medium. As shown in Figure 4a, the average H_2_O_2_ production rate of the parent NQ‐COF_BD_ was 2416 µmol g^−1^ h^−1^, whereas after introducing the POD site, NQ‐COF_BD_‐O showed a significant increase to 4569 µmol g^−1^ h^−1^, a 1.89‐fold improvement, directly demonstrating that the POD site significantly enhanced photocatalytic activity by optimizing charge dynamics. When isopropyl alcohol (IPA) was used as a hole scavenger, the rates of the two catalysts further increased to 7,060 and 10,189 µmol g^−1^ h^−1^, respectively, with the latter exceeding 10 mmol g^−1^ h^−1^, demonstrating excellent catalytic potential (Figure 4g). Under monochromatic light, the apparent quantum yield (AQY) of NQ‐COF_BD_‐O at 460 nm reached 6.1%, surpassing most reported metal‐free COF photocatalysts. The AQY decreased with increasing wavelength, consistent with the material's visible absorption spectrum, indicating that the H_2_O_2_ production process is dominated by bandgap transitions (Figures 4b and S29). Compared with representative COF‐based photocatalysts (Figure 4d and Table S4), NQ‐COF_BD_‐O demonstrates leading performance under sacrificial‐agent‐free conditions, highlighting the innovation and advanced nature of this work. To evaluate its application potential, we tested the photocatalytic performance of NQ‐COF_BD_‐O in various real‐world water samples (Figure 4c). The yield in tap water increased to 7013 µmol g^−1^ h^−1^, which is 1.54 times that in pure water, attributed to the presence of trace inorganic salt ions (such as (Na^+^, Cl^−^, K^+^, etc.) in tap water; these ions act as electrolytes, significantly enhancing charge transport at the liquid‐solid interface of the COF material and promoting the efficient separation of electron‐hole pairs, thereby improving the overall photocatalytic efficiency [47, 48]. In complex seawater, river water, and lake water, the yields were 3455, 4197, and 3327 µmol g^−1^ h^−1^, respectively, all higher than the parent material's 2416 µmol g^−1^ h^−1^ in pure water; the slight decrease in performance in seawater and lake water stems from partial surface coverage and corrosion caused by high concentrations of ions and organic pollutants [38]. The above results fully confirm that NQ‐COF_BD_‐O possesses the ability to efficiently produce H_2_O_2_ in complex real‐world aquatic environments. To further evaluate its pH adaptability, we tested the photocatalytic H_2_O_2_ production performance at representative pH values (3, 5, 7, 9, and 11) (Figure S30). The results demonstrated that NQ‐COF_BD_‐O exhibited remarkable activity across this wide pH range, maintaining high H_2_O_2_ yields even under strongly acidic and alkaline conditions, thus confirming its excellent pH adaptability and promising potential for practical applications.

Photocatalytic H_2_O_2_ generation and in situ decomposition are competitive processes [38, 49]. Kinetic experiments (Figures S31 and S32) showed that the formation rate constant (K_f_) of NQ‐COF_BD_‐O was significantly higher than that of NQ‐COF_BD_; in contrast, the decomposition rate constants (K_d_) of both materials were extremely low, with the H_2_O_2_ decomposition percentage remaining below 10% under visible‐light irradiation for 2 h. This confirms that the quinoline‐linked COF framework possesses excellent photo‐oxidation resistance and does not catalyze H_2_O_2_ decomposition. The combination of a high formation rate and a low decomposition rate is key to the high H_2_O_2_ yield of NQ‐COF_BD_‐O. Five consecutive cycling tests (1 h each) of NQ‐COF_BD_‐O showed no significant decay in photocatalytic H_2_O_2_ production activity (Figures 4e and S33). Meanwhile, the XRD, FT‐IR, SEM, and XPS characterization results of the samples after continuous illumination for 12 h revealed that both the crystal structure and microstructure of the material remained intact, and the N^+^─O^−^ bond is completely preserved (Figures S34–S37), demonstrating excellent structural and photochemical stability suitable for long‐term photocatalytic reactions.

### 2.4 Mechanistic Insights Into POD‐Facilitated Photocatalysis

To elucidate the dominant reaction pathways and key active intermediates in the photocatalytic production of H_2_O_2_, we conducted atmosphere‐controlled and radical‐trapping experiments (Figure 4f,g). Upon gradually reducing the partial pressure of O_2_, the yield of NQ‐COF_BD_‐O dropped sharply from 4569 µmol g^−1^ h^−1^ to 1786 (air) and 611 (N_2_); the parent material NQ‐COF_BD_ exhibited a consistent trend, with yields under air and N_2_ conditions amounting to 45.9% and 19.6% of those under pure oxygen, respectively, confirming that ORR is the predominant pathway, while a minor contribution from WOR cannot be completely ruled out given the detectable H_2_O_2_ production of 611 µmol g^−1^ h^−1^ under N_2_ (approximately 13% of that under O_2_). Radical trapping experiments showed that the addition of 10 mM AgNO_3_ (an e^−^ scavenger) or p‐BQ (a ·O_2_^−^ scavenger) suppressed the yields of both materials to extremely low levels; under N_2_ atmosphere, the yield was nearly zero upon AgNO_3_ addition, indicating that photo‐generated electrons and ·O_2_^−^ are indispensable intermediates, and the reaction follows an indirect two‐electron ORR pathway (O_2_ → ·O_2_^−^ → H_2_O_2_). The two materials exhibited starkly different responses to ^1^O_2_: upon addition of 10 mM ^1^O_2_ scavenger L‐histidine, the yield of NQ‐COF_BD_ decreased significantly by 63%, whereas that of NQ‐COF_BD_‐O remained essentially unchanged. This difference reveals that in the parent material, a large amount of ·O_2_^−^ is oxidized by holes to form ^1^O_2_ (·O_2_^−^ + h^+^ → ^1^O_2_), which serves as a critical intermediate that is further converted to H_2_O_2_ [50]. In contrast, in NQ‐COF_BD_‐O, although a small amount of ^1^O_2_ is still generated, it acts as an unproductive byproduct that does not participate in the main reaction pathway. The POD site redirects the dominant conversion of ·O_2_^−^ to the direct two‐electron ORR route, thus bypassing the hole‐mediated ^1^O_2_ intermediate pathway. The addition of 10 vol% of the hole scavenger IPA increased the yields of both products by more than twofold, further confirming that hole recombination is the key factor limiting the activity of the parent material, while the POD site effectively alleviates this issue by promoting spatial separation of charges.

**FIGURE 5:**
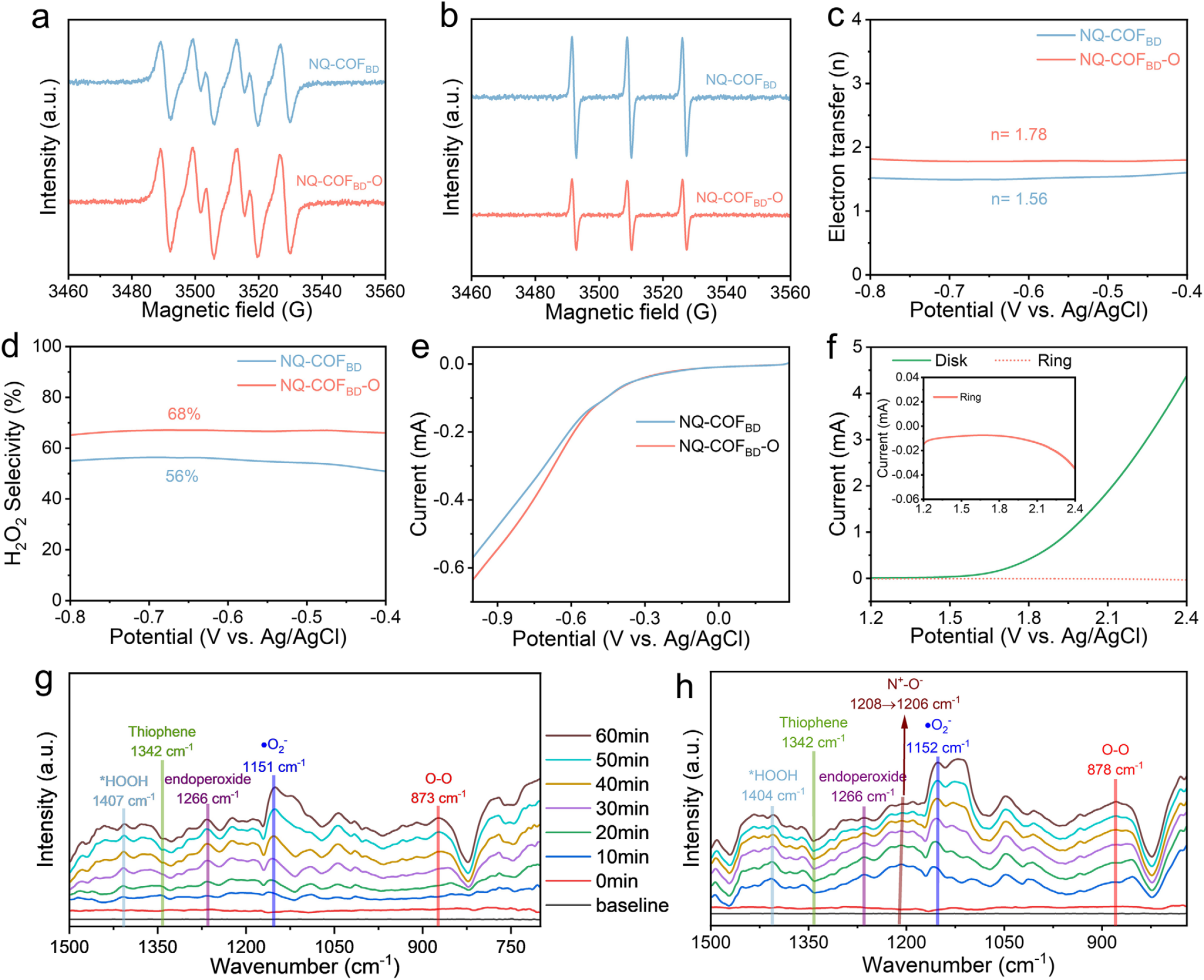
(a) The generation of ·O_2_^−^ on NQ‐COF_BD_ and NQ‐COF_BD_‐O was monitored via EPR spectroscopy, with the respective species trapped by DMPO. (b) The generation of ^1^O_2_ on NQ‐COF_BD_ and NQ‐COF_BD_‐O was monitored via EPR spectroscopy, with the respective species trapped by TEMP. (c) Electron transfer number (n) and (d) H_2_O_2_ selectivity of NQ‐COF_BD_‐O and NQ‐COF_BD_ were calculated on the basis of RRDE. (e) Polarization curves recorded at 1000 rpm in the RRDE test system for NQ‐COF_BD_ and NQ‐COF_BD_‐O in 0.1 M phosphate‐buffered saline. (f) RRDE voltammograms of NQ‐COF_BD_‐O with a potential of −0.23 V vs. Ag/AgCl on a Pt ring electrode to detect O_2_. In situ DRIFTS spectra of (g) NQ‐COF_BD_ and (h) NQ‐COF_BD_‐O during H_2_O_2_ photosynthesis over 60 min.

Based on preliminary inferences from the aforementioned reaction pathways, we systematically characterized the key reactive oxygen species by combining in situ EPR spectroscopy with probe experiments. Using DMPO as the spin‐trapping agent, characteristic DMPO‐·O_2_^−^ signals were detected in both materials under visible light (Figure 5a), and the signal intensity of NQ‐COF_BD_‐O was significantly higher than that of NQ‐COF_BD_, indicating that the POD site promotes the generation of ·O_2_^−^. When TEMP was used to capture ^1^O_2_, both materials exhibited TEMP‐^1^O_2_ signals, but the signal from NQ‐COF_BD_ was stronger, consistent with the results of the capture experiments (Figures 5b andS38). When TEMPO was used to trap h^+^, the signal from NQ‐COF_BD_ was weaker than that from NQ‐COF_BD_‐O, and its own ·O_2_^−^ signal decreased by 52%, whereas that of NQ‐COF_BD_‐O decreased by only 39%. Combined with the ^1^O_2_ scavenging experiment, the ^1^O_2_ generated on NQ‐COF_BD_‐O does not participate in the main reaction and is an ineffective byproduct (O_2_ + e^−^ → ·O_2_^−^, ·O_2_^−^ + h^+^ → ^1^O_2_); whereas NQ‐COF_BD_ consumes more holes to generate ^1^O_2_, which is further converted to H_2_O_2_ (Figure S39). The ·OH signal was weak and likely originated from the self‐decomposition of H_2_O_2_. Quantitative analysis showed that the ·O_2_^−^ production rate of NQ‐COF_BD_‐O, as measured by the NBT probe, was 65.98 µmol·L^−1^·h^−1^, which is 2.04 times that of the parent compound (32.36 µmol·L^−1^·h^−1^) (Figure S40) [51, 52, 53, 54]. The ^1^O_2_ generation rate detected by the ABDA probe was higher for NQ‐COF_BD_, consistent with the above conclusion (Figure S41) [55, 56]. In summary, the POD site enhances the ·O_2_^−^ generation efficiency by a factor of 2.04 and, by suppressing its inefficient conversion to ^1^O_2_, enables the directed conversion of charge carriers to H_2_O_2_.

To quantitatively verify the electron transfer pathways and kinetic processes, we performed rotating ring‐disk electrode (RRDE) measurements. The average number of electron transfers (n) for NQ‐COF_BD_ and NQ‐COF_BD_‐O were measured to be 1.56 and 1.78, respectively (Figure 5c,d). Both values are below 2.0, which is attributed to the partial consumption of ·O_2_^−^ by holes to generate ^1^O_2_; the value for NQ‐COF_BD_‐O is significantly closer to 2, confirming that it enhances the selectivity for the two‐electron ORR by suppressing ^1^O_2_ generation. In an O_2_‐saturated 0.1 M PBS solution, the onset potentials for the 2e^−^ ORR of NQ‐COF_BD_ and NQ‐COF_BD_‐O were 0.22 and 0.17 V, respectively (relative to Ag/AgCl, Figure 5e), with NQ‐COF_BD_‐O exhibiting a 50 mV lower overpotential, confirming its faster 2e^−^ ORR kinetics [57, 58, 59]. RRDE measurements under argon atmosphere confirmed that the contribution of water oxidation to hydrogen peroxide generation is negligible (Figures 5f and S42 and S43), consistent with the conclusions from atmospheric control experiments and EPR experiments.

To visually verify the two‐step single‐electron ORR process and capture the evolution of reaction intermediates in real time, we used in situ diffuse reflectance infrared Fourier transform spectroscopy (DRIFTS) to dynamically monitor the photocatalytic reaction process. With water vapor and visible‐light irradiation in O_2_‐saturated atmosphere, the vibrational bands assigned to adsorbed O─O (873 and 878 cm^−1^), ·O_2_^−^ (1151 and 1152 cm^−1^), *OOH intermediate (1266 cm^−1^), and HOOH (1404 and 1407 cm^−1^) all grew progressively with irradiation time (Figure 5g,h). This unambiguous spectral evidence confirms the stepwise O_2_ → ·O_2_^−^ → *OOH → H_2_O_2_ pathway [60, 61]. Crucially, the N^+^─O^−^ bond vibration of the POD site at 1208 cm^−1^ underwent a distinct red‐shift to 1206 cm^−1^ over the course of the reaction, demonstrating that the POD site actively participates in the catalytic cycle and serves as the genuine active center for sustained H_2_O_2_ production.

**FIGURE 6:**
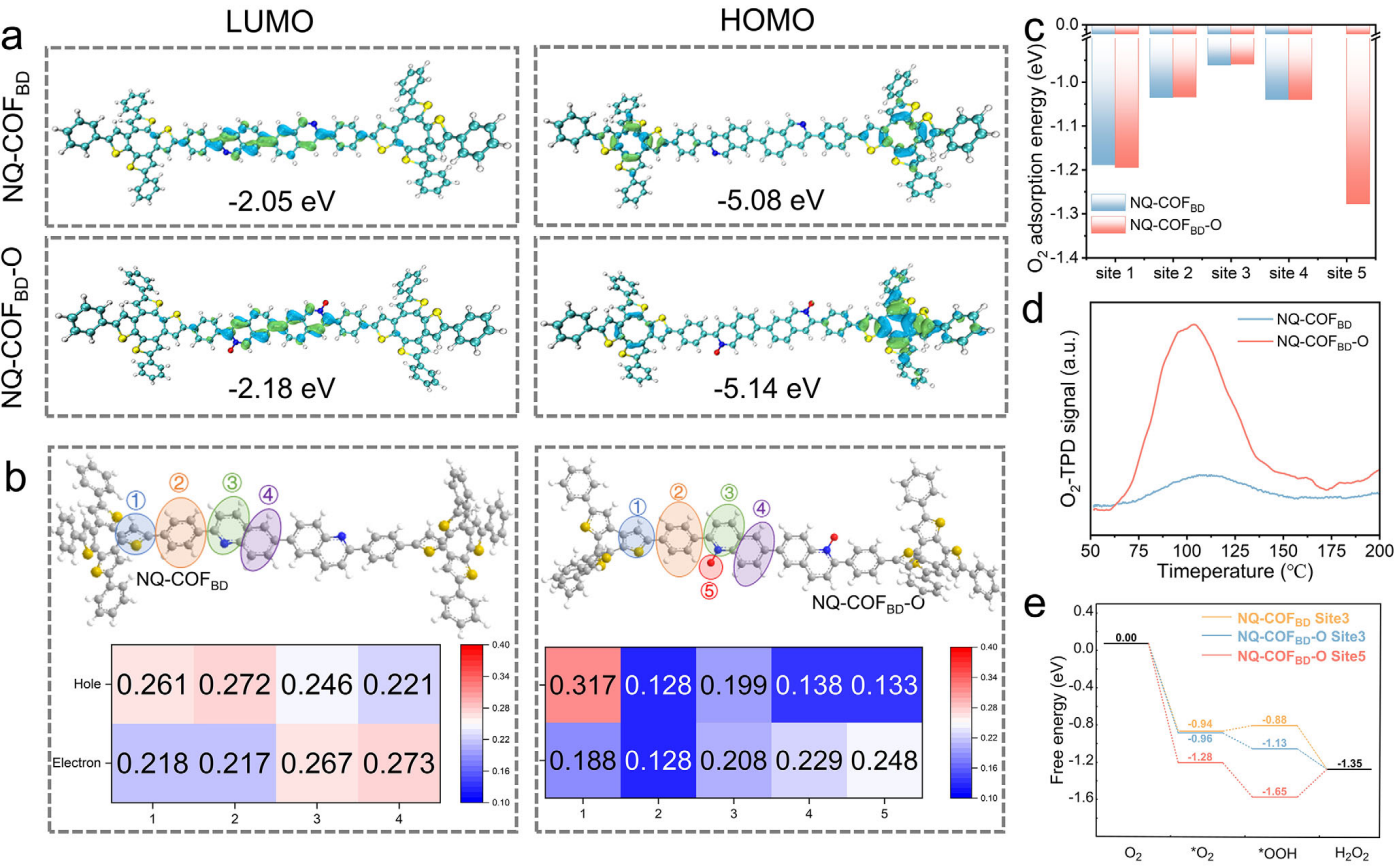
(a) The HOMO and LUMO orbital distributions of the simplified NQ‐COF_BD_ and NQ‐COF_BD_‐O segments based on DFT calculations. (b) Hole‐electron distribution heat map of each fragment in the NQ‐COF_BD_ and NQ‐COF_BD_‐O molecules in the first excited singlet state (S_1_). (c) The oxygen molecule adsorption energy at the representative active sites of NQ‐COF_BD_ and NQ‐COF_BD_‐O. (d) O_2_‐TPD measurements of NQ‐COF_BD_ and NQ‐COF_BD_‐O. (e) Gibbs free energy diagrams of ORR of the NQ‐COF_BD_ and NQ‐COF_BD_‐O.

To elucidate the microscopic origin of the enhanced ORR performance, we systematically analyzed the excited‐state charge distribution, oxygen adsorption, and reaction pathways using density functional theory (DFT) calculations. To ensure the robustness of our theoretical predictions, we established a direct cross‐validation with our experimental results: the calculated narrowing of the HOMO‐LUMO energy gap is consistent with the optical bandgap reduction observed by UV–vis DRS; the charge separation efficiency indicated by the computed electron‐hole distributions is corroborated by the significant reduction in exciton binding energy measured via temperature‐dependent PL spectroscopy (from 44.7 to 20.3 meV); and the improved O_2_ adsorption predicted by the model is further confirmed by the larger O_2_ desorption signals in the O_2_‐TPD experiments. Frontier orbital analysis of simplified structural units (Figure 6a) revealed that the HOMO and LUMO of both NQ‐COF_BD_ and NQ‐COF_BD_‐O exhibit pronounced spatial separation, providing an intrinsic structural basis for charge separation. Notably, the frontier orbital energy gap of NQ‐COF_BD_‐O is significantly narrower than that of the parent material, in full agreement with the optical bandgap narrowing observed by UV–vis DRS. To identify the ORR active sites and excited‐state charge localization, we calculated electron–hole distribution heatmaps for the first singlet excited state (S_1_) (Figure 6b). The POD sites create a spatially decoupled redox dual‐center architecture: Site 1 exhibits strong hole enrichment (hole density 0.317), acting as the oxidation center for water oxidation; Sites 4 (electron density 0.229) and 5 (electron density 0.248) serve as electron‐rich reduction centers that dominate the ORR. Crucially, Site 2 shows nearly balanced and suppressed charge densities (hole 0.128, electron 0.128), effectively blocking bulk electron–hole recombination and enabling precise functional zoning of catalytic sites. Consistently, O_2_ adsorption energy calculations show that the terminal oxygen of the N^+^─O^−^ bond (Site 5) in NQ‐COF_BD_‐O exhibits an adsorption energy of −1.278 eV, which is 0.089 eV stronger than that of the optimal Site 1 in NQ‐COF_BD_ (−1.189 eV), directly confirming that the POD site is the dedicated O_2_ adsorption and activation center (Figures 6c and S44 and S45).

To verify the conclusions regarding active sites and adsorption energies derived from theoretical calculations, we conducted O_2_‐TPD measurements and, in conjunction with Gibbs free energy analysis, determined the energy barrier changes for the rate‐determining step of the ORR. The results show that the peak area for the desorption of physically adsorbed oxygen on NQ‐COF_BD_‐O in the 50°C–150°C range is significantly larger than that on NQ‐COF_BD_, directly demonstrating that the POD sites significantly enhance oxygen capture capacity, which is fully consistent with the computational results (Figure 6d). Further analysis of the Gibbs free energy plots indicated that the rate‐determining step of the ORR process is the formation of the OOH intermediate: at position 5 of NQ‐COF_BD_‐O, the formation energies of O_2_ and the OOH intermediate at position 5 of NQ‐COF_BD_‐O are −1.28 and −1.65 eV, respectively, both significantly lower than those at position 3 of NQ‐COF_BD_‐O (−0.96 and −1.13 eV) and at position 3 of NQ‐COF_BD_ (−0.94 and −0.88 eV) (Figure 6e); In particular, the formation energy of the OOH intermediate is 0.77 eV lower than that of the parent compound, significantly reducing the kinetic energy barrier for the ORR reaction. In summary, the site‐selective oxidation of the nitrogen atom in the quinoline ring, achieved by introducing a permanent nitrogen cation–oxygen anion pair, on the one hand, reconfigures the electronic structure of the framework, induces a strong built‐in electric field, and constructs a spatially decoupled redox bicentric sites, thereby enabling the efficient separation and directed transport of photogenerated charge carriers; on the other hand, it transforms the terminal oxygen atom of the N^+^─O^−^ pair into a dedicated center for O_2_ adsorption and activation, significantly lowering the energy barrier of the rate‐determining step of the ORR and ultimately enabling the highly efficient and highly selective generation of H_2_O_2_.

### 2.5 Validation of the Feasibility of POD Engineering in Quinoline COFs

**FIGURE 7:**
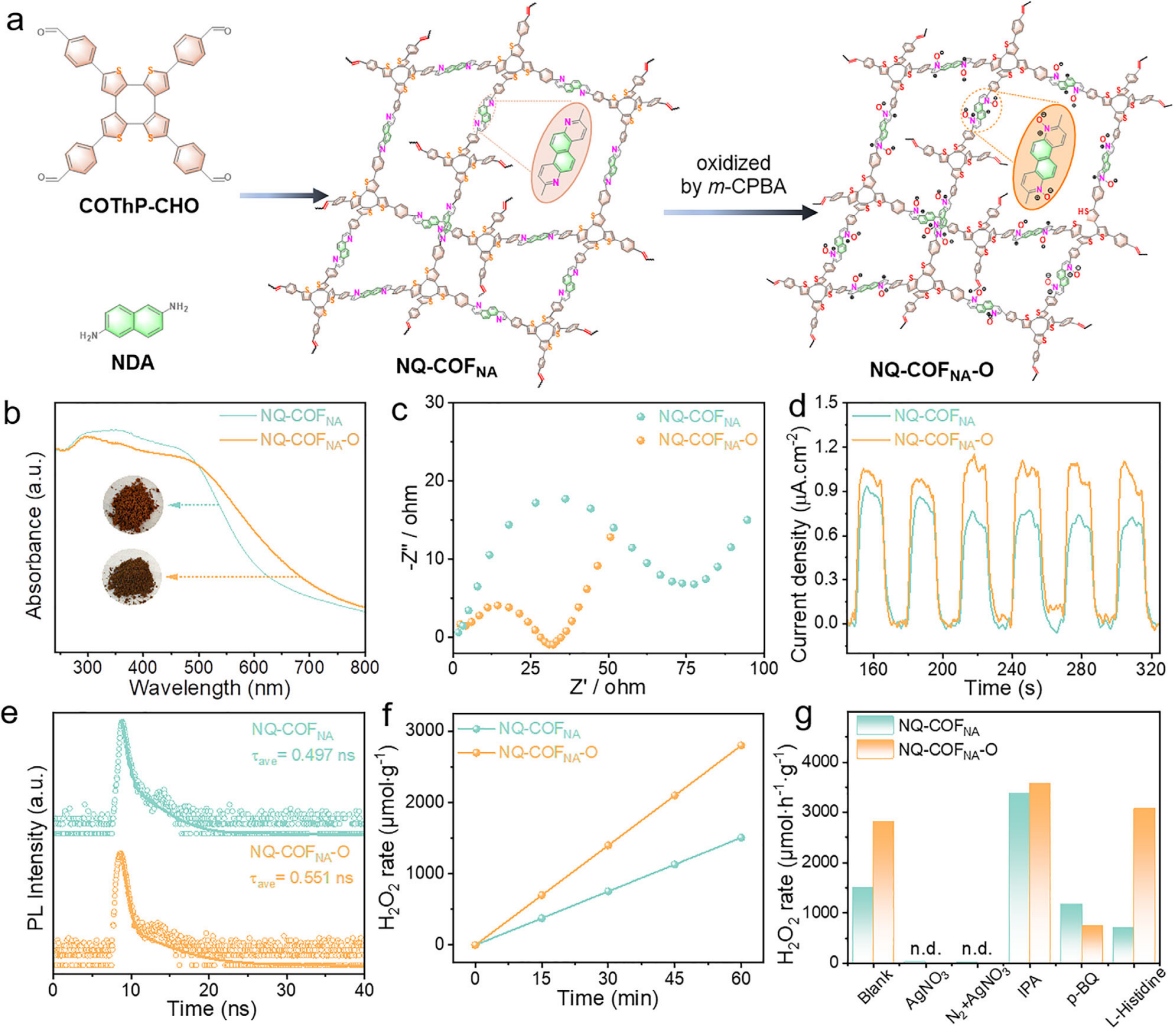
(a) Synthesis and structures, (b) The UV–vis DRS, (c) Electrochemical impedance spectroscopy (EIS) Nyquist plots, (d) Comparison of the photocurrent response, (e) Excited‐state lifetime measurement of NQ‐COF_NA_ and NQ‐COF_NA_‐O through TCSPC. (f) Time‐dependent H_2_O_2_ production curves of all the NQ‐COF_NA_ and NQ‐COF_NA_‐O under visible‐light irradiation over 1 h. (g) Photocatalytic H_2_O_2_ generation using NQ‐COF_NA_ and NQ‐COF_NA_‐O under different sacrificial agents.

To evaluate whether the enhancement of photocatalytic H_2_O_2_ production performance by permanent oxidative dipole (POD) sites in quinoline‐linked COFs can be extended to other systems, we selected naphthalene‐2,6‐diamine (NDA) as a replacement for 4,4′‐biphenyldiamine and constructed a control model system, NQ‐COF_NA_, and its oxidized derivative NQ‐COF_NA_‐O, using exactly the same one‐pot three‐component [4+2] annulation and mild post‐oxidation procedures as those employed for NQ‐COF_BD_ (Figure 7a). The generality of the strategy was systematically validated in terms of structure, photoelectric properties, catalysis, and mechanism.

Structural characterization results fully confirmed that POD sites can be site‐specifically, efficiently, and non‐destructively introduced into the NQ‐COF_NA_ framework. FT‐IR, XPS, PXRD, and pore structure analyses indicated that both NQ‐COF_NA_ and NQ‐COF_NA_‐O maintained high crystallinity and typical porous COF characteristics (Figures S46–S54 and Table S3). After oxidation, a characteristic N^+^─O^−^ bond stretching vibration peak appeared at 1211 cm^−1^, directly demonstrating the successful construction of POD sites without destroying the crystal structure or microscopic morphology of the COF and the calculated oxidation rate was 86.5%. (Figures S55–S58). The evolution of photoelectric properties was highly consistent with that observed for the NQ‐COF_BD_ system, further confirming the consistent role of the POD modulation mechanism. The POD sites substantially increased the molecular dipole moment of NQ‐COF_NA_ from 0.09 Debye to 0.36 Debye (a 4.0‐fold enhancement), markedly strengthening the built‐in electric field within the framework. Simultaneously, the POD sites induced bandgap narrowing, significantly reduced charge transfer resistance, extended the carrier lifetime from 0.497 to 0.551 ns, and improved hydrophilicity (Figures 7b–e and S59–S61). These results demonstrate the consistent effectiveness of the triple synergistic mechanism of POD sites: enhancing the built‑in electric field, reducing exciton binding energy, and strengthening oxygen adsorption/activation.

Photocatalytic performance tests demonstrated the generality of this strategy. Under sacrificial‐agent‐free, O_2_‐saturated, visible‐light (λ ≥ 420 nm) conditions, the H_2_O_2_ production rate of NQ‐COF_NA_‐O reached 2803 µmol g^−1^ h^−1^, representing a 1.86‐fold enhancement over the parent NQ‐COF_NA_ (1508 µmol g^−1^ h^−1^), in excellent agreement with the 1.89‐fold enhancement observed for the NQ‐COF_BD_ system. The apparent quantum efficiency (AQY) of NQ‐COF_NA_‐O at 460 nm reached as high as 9.97%, and the material exhibited excellent catalytic activity and cycling stability in real water matrices (Figures 7f and S62–S70). Mechanistic studies further confirmed that NQ‐COF_NA_‐O follows exactly the same photocatalytic pathway as NQ‐COF_BD_‐O: the indirect two‐electron oxygen reduction (2e^−^ ORR) is the dominant route, and ^1^O_2_ does not participate in the main reaction (Figure 7g). DFT calculations reveal that this system also forms a spatially decoupled redox dual‐center structure featuring the N^+^─O^−^ group as the reduction center and the COTh ring as the oxidation center (Figures S71–S76). In summary, the permanent oxidative dipole (POD) post‐modification strategy proposed in this work demonstrates consistent effectiveness in quinoline‐linked COF systems with different aromatic amine units, stably achieving a substantial enhancement in photocatalytic H_2_O_2_ production performance by a factor of approximately 1.86–1.89. This work provides an efficient and promising paradigm for the rational design of high‐performance COF‐based photocatalysts.

## 3 Discussion

In summary, we have developed a mild and site‐selective post‐oxidation strategy to precisely implant permanent oxidative dipole (POD) sites, featuring N^+^─O^−^ charge pairs, into the framework of a three‐dimensional crystalline quinoline COF (NQ‐COF_BD_). The resulting NQ‐COF_BD_‐O effectively overcomes two long‐standing kinetic bottlenecks, high exciton binding energy and intrinsically weak oxygen activation, by fundamentally disrupting the originally delocalized electron distribution within the skeleton. The localized charge polarization induced by the POD sites exerts a triple synergistic modulation: a 1.9‐fold enhancement in the molecular dipole moment, a 54.6% reduction in exciton binding energy, and a 1.26‐fold increase in surface potential. Consequently, the built‐in electric field is notably strengthened, promoting the efficient dissociation of excitons into free carriers at the microscopic scale. Crucially, the POD sites act as dedicated oxygen adsorption and activation centers, forming spatially decoupled dual‐center reaction sites with the framework. This configuration drastically lowers the energy barrier of the rate‐determining ORR step (*OOH formation) by 0.77 eV, while concurrently suppressing the detrimental conversion of superoxide radicals into singlet oxygen. Driven by this synergistic mechanism, NQ‐COF_BD_‐O achieves a remarkable H_2_O_2_ production rate of 4569 µmol g^−1^ h^−1^ in a sacrificial‐agent‐free system (a 1.89‐fold enhancement), and this dipole engineering strategy is further validated in another representative quinoline COF system, yielding a similar 1.86‐fold enhancement. Beyond offering a new paradigm for dipole engineering rooted in the reconstruction of the intrinsic electronic structure, this work elucidates an effective design principle, the precise construction of localized charge gradients, to concurrently resolve the enduring challenges of exciton dissociation and surface reaction kinetics.

## Author Contributions

**Yong‐Quan Wu**: writing – review and editing, funding acquisition, writing – original draft, conceptualization, investigation, validation, project administration, methodology. **Zhi‐Bo Zuo**: investigation, validation. **Wei‐Rong Cui**: conceptualization, writing – review and editing, project administration, funding acquisition, investigation, validation, writing – original draft, methodology.

## Conflicts of Interest

The authors declare no conflicts of interest.

## Supporting information




**Supporting File**: advs77745‐sup‐0001‐SuppMat.docx.

## Data Availability

The data that support the findings of this study are available on request from the corresponding author. The data are not publicly available due to privacy or ethical restrictions.
