# Supplementary material for: Permanent Oxidative Dipole Engineering in Quinoline COFs for Boosting Exciton Dissociation and Oxygen Activation in H2O2 Photosynthesis
Source: Adv Sci (Weinh). 2026 Sep 16:e77745. Online ahead of print. doi: 10.1002/advs.77745 (PMC13583104; doi:10.1002/advs.77745)
Supplement: Supplementary file 1 [file ADVS-9999-e77745-s001.docx]

**Supporting Information for**

**Permanent oxidative dipole engineering in quinoline COFs for boosting exciton dissociation and oxygen activation in H_2_O_2_ Photosynthesis**

Yong-Quan Wu, Zhi-Bo Zuo, Wei-Rong Cui*

College of Chemistry and Materials, Gannan Normal University, Ganzhou 341000, China

E-mail: wrcui@gnnu.edu.cn (W.-R. Cui).

**Table of Contents**

**Part I: Materials and Methods**‧‧‧‧‧‧‧‧‧‧‧‧‧‧‧‧‧‧‧‧‧‧‧‧‧‧‧‧‧‧‧‧‧‧‧‧‧‧‧‧‧‧‧‧‧‧‧‧‧‧‧‧‧‧‧‧‧‧‧‧‧‧S2

**Part II: General Synthesis Methods**‧‧‧‧‧‧‧‧‧‧‧‧‧‧‧‧‧‧‧‧‧‧‧‧‧‧‧‧‧‧‧‧‧‧‧‧‧‧‧‧‧‧‧‧‧‧‧‧‧‧‧‧S10

**Part III: Supplementary Charts and Tables**‧‧‧‧‧‧‧‧‧‧‧‧‧‧‧‧‧‧‧‧‧‧‧‧‧‧‧‧‧‧‧‧‧‧‧‧‧‧S17

**References**‧‧‧‧‧‧‧‧‧‧‧‧‧‧‧‧‧‧‧‧‧‧‧‧‧‧‧‧‧‧‧‧‧‧‧‧‧‧‧‧‧‧‧‧‧‧‧‧‧‧‧‧‧‧‧‧‧‧‧‧‧‧‧‧‧‧‧‧‧‧‧‧‧‧‧‧‧‧‧‧‧‧‧‧‧‧‧‧‧‧‧‧‧S55

**Part I: Materials and Methods**

**1.1 Reagents**

Common solvents such as toluene, dichloromethane (DCM), petroleum ether, ethyl acetate (EA), tetrahydrofuran (THF), mesitylene, ethanol, chloroform, N,N-dimethylformamide (DMF), 1,4-dioxane, tert-butyl peroxide, triethylamine, and reagents such as 2,4-dibromothiophene, palladium acetate (Pd(OAc)_2_), silver carbonate, bis(pinacol)boronic acid, 4-formylphenylboronic acid, tetra(triphenylphosphine)palladium(IV) (Pd(PPh_3_)_4_), [1,1'-bis(diphenylphosphino)ferrocene]palladium(II) dichloride (PdCl_2_(dppf)), naphthalene-2,6-diamine, and biphenylamine were all purchased from J&K, Macklin, and Innochem. All were of analytical grade and were used directly without further purification.

**1.2 Characterization methods**

¹H NMR (400 MHz) and ¹³C NMR (100 MHz) spectra were acquired on a Bruker Avance series spectrometer using tetramethylsilane (TMS) as the internal standard. Peak types are denoted as follows: s (single peak), d (double peak), t (triple peak). High-resolution mass spectrometry (MS) was performed using a Bruker maxis ultra-high-resolution time-of-flight mass spectrometer. Matrix-assisted laser desorption/ionization time-of-flight mass spectrometry (MALDI-TOF MS) was obtained using an AB 5800 system. Powder X-ray diffraction (PXRD) data for nanomaterials were acquired on a Bruker AXS D8 Advance A25 powder X-ray diffractometer (40 kV, 40 mA) using Cu Kα radiation (λ = 1.5406 Å). Fourier-transform infrared (FT-IR) spectra were recorded on a Nicolet Impact 410 FT-IR spectrometer. X-ray photoelectron spectroscopy (XPS) was performed on a Thermo Scientific K-Alpha spectrometer. Material morphology was characterized by scanning electron microscopy (SEM, JEM-2010, JEOL). Solid-state 13C cross-polarized magic-angle spinning (^13^C CP/MAS NMR) spectra were recorded using a 4 mm dual-resonance MAS probe, with a sample rotation rate of 10.0 kHz, a contact time of 2 ms (ramp 100), and a pulse delay of 3 s; samples were degassed at 120 °C for 12 h prior to testing. N₂ adsorption–desorption isotherms were measured at 77 K using a Micromeritics ASAP 2020M system; the specific surface area was calculated according to the Brunauer–Emmett–Teller (BET) model, and the pore size distribution curve was obtained using the non-local density functional theory (NLDFT) method. UV-visible diffuse reflectance spectroscopy (DRS) was recorded at room temperature using a PE Lambda 900 UV–Vis spectrophotometer. Electron paramagnetic resonance (EPR) spectra were measured at room temperature using a Bruker ELEXSYS II E 500 EPR spectrometer. In-situ diffuse reflectance infrared Fourier transform spectroscopy (DRIFTS) measurements were performed using a Nicolet iS50 spectrometer (Thermo, USA).

**1.3 Electrochemical Testing**

The Mott–Schottky plots, photocurrent responses, and electrochemical impedance of the catalyst were measured using an electrochemical workstation (CHI660E, CHI Instruments, Shanghai, China). A 300 W xenon lamp was used as the light source (λ > 420 nm, average intensity: 100 mW·cm^-2^), and a 0.5 M Na_2_SO_4_ aqueous solution (pH = 6.5) was used as the supporting electrolyte during photocurrent measurements. An Ag/AgCl electrode served as the reference electrode, and a platinum wire served as the counter electrode. A uniform suspension was prepared by sonically treating 20.0 μL of Nafion (5 wt%, dispersed in water), 0.5 mL of anhydrous ethanol, and 2.0 mg of COF for 1 hour. Then, 200 μL of the suspension was dispensed onto an ITO glass substrate and dried at room temperature. The applied potential was converted to the RHE potential relative to Ag/AgCl using the following equation:

E (VS. RHE) = E (VS. Ag/AgCl) + 0.197V + 0.0591 * pH

**1.4 Photocatalytic Production of H_2_O_2_**

3 mg of photocatalyst and 45 mL of deionized water were uniformly dispersed by ultrasonication and added to the photocatalytic reaction apparatus. Prior to the reaction, O₂ was bubbled into the mixture for 30 minutes, followed by a 30-minute reaction in the dark; thereafter, O₂ was continuously bubbled into the suspension throughout the photocatalytic process. The light source was a 300 W xenon lamp (PLS-SXE300C, Perfect Light) equipped with a cutoff filter (λ > 420 nm). Every 15 minutes, 1.0 mL of the reaction solution was filtered through a 0.22 μm filter to remove the photocatalyst. Add 0.5 mL of the filtered reaction solution to 2 mL of a color developer (consisting of 0.4 M potassium iodide (KI) solution and 1 mL of 0.1 M potassium hydrogen phthalate (C_8_H_5_KO_4_) solution). After allowing the mixture to stand for 1 hour, measure the concentration of hydrogen peroxide generated by the reaction using a UV-visible spectrophotometer.

Standard curve for the iodometric colorimetric analysis: Mix 1 mL of 0.4 M potassium iodide (KI) solution, 1 mL of 0.1 M potassium hydrogen phthalate (C_8_H_5_KO_4_) solution, and 1 mL of a known hydrogen peroxide (H_2_O_2_) solution of varying concentrations thoroughly, and let stand for 60 min. Under acidic conditions, H_2_O_2_ reacts with I⁻ to form I₃⁻. As detected by UV spectrophotometry, I₃⁻ exhibits a strong absorption peak at approximately 350 nm. Figure S1 shows the linear relationship between the concentration of the H_2_O_2_ solution and the intensity of the UV absorption peak.

$$3I^{-}+H_{2}O_{2}+2H^{+}\to I_{3}^{-}+H_{2}O$$

Figure S1. (a) Ultraviolet absorption spectrum of iodometric method; (b) Standard curves of H_2_O_2_ concentration and ultraviolet absorption peak intensity.

Standard curve for the titanate colorimetric method: The titanate working solution is prepared fresh by mixing 0.1 mol/L titanate stock solution and 2 mol/L H_2_SO_4_ in a 1:4 (v/v) ratio. Mix 1 mL of the titanate working solution with 2 mL of a hydrogen peroxide (H_2_O_2_) solution of a known concentration, and let stand for 10 minutes. Under acidic conditions, H_2_O_2_ reacts with Ti⁴⁺ to form the titanium peroxide complex [TiO(H_2_O_2_)]^2+^. As detected by UV spectrophotometry, [TiO(H_2_O_2_)]²⁺ exhibits a strong absorption peak at approximately 405 nm. Figure S2 shows the linear relationship between the concentration of the H_2_O_2_ solution and the intensity of the UV absorption peak.

Figure S2. (a) Ultraviolet absorption spectrum of the titanium sulfate method; (b) Standard curve of hydrogen peroxide concentration versus the intensity of the ultraviolet absorption peak.

The effects of different conditions (air, darkness, and N_2_) on the photocatalytic H₂O₂ production performance in aqueous solutions were investigated. Control experiment under N₂ atmosphere: Prior to the photocatalytic reaction, nitrogen gas was bubbled into the mixed solution for 30 minutes, followed by a 30-minute dark reaction; nitrogen gas was then continuously bubbled into the suspension throughout the entire photocatalytic process. Under the same oxygen-supply conditions, the effects of different scavengers on the photocatalytic H_2_O_2_ production performance were investigated. The ratio of tert-butanol to water was 1:9, the ratio of isopropanol to water was 1:9, the concentration of AgNO_3_ was 10 mM, the concentration of p-BQ was 10 mM, and the concentration of L-histidine was 10 mM. For experiments using other aqueous solutions, pure water was replaced with the respective solution. For samples exposed to light in seawater and those using p-BQ as the sacrificial agent, hydrogen peroxide was detected using the titanium sulfate method; for all other samples, the iodine volumetric method was used.

**1.5 Nitroblue tetrazolium (NBT) colorimetric assay for ‧O_2_^-^**

The concentration of **‧**O_2_^-^ was determined using the NBT colorimetric method and a UV spectrophotometer. 3 mg of COFs and 25 mL of NBT solution (0.1 mM) were thoroughly mixed by ultrasonication. A 300 W xenon lamp (λ > 420 nm) was used as the light source. After different exposure times (0 min, 15 min, 30 min, 45 min, 60 min, 75 min, 90 min, 105 min, 120 min), the photocatalyst was filtered out of 2 mL of the post-reaction solution using a 0.22 μm pore size filter membrane. Using the NBT degradation method, changes in absorbance were monitored at a specific wavelength of 259 nm using a UV-Vis spectrophotometer to detect ‧O_2_^-^ generated during the photocatalytic reaction. Given that the molar ratio of the reaction between NBT and O_2_ is 1:4 (Figure S3), the generation of ‧O_2_^-^ was quantified by measuring the change in absorbance of the characteristic NBT signal at 259 nm. Therefore, the concentration of ‧O_2_^-^ is determined by calculating the change in NBT concentration resulting from degradation by the COF samples at different time points.

**Figure S3.** Reaction equation between NBT and ‧O_2_^-^ (molar ratio = 1:4).

**1.6 9,10-Araconiyl-bis(methylenedi)dipropionic acid (ABDA) colorimetric method for detecting ^1^O_2_**

The concentration of ^1^O_2_ was detected using the ABDA colorimetric method and a UV-Vis spectrophotometer. A mixture of 3 mg COFs, 50 mL PBS solution (0.1 mM), and 1 mL ABDA solution (5 mM) was sonicated to ensure homogeneity. A 300 W xenon lamp (λ > 420 nm) served as the light source. After irradiation for different durations (0 min, 1 min, 2 min, 3 min, 4 min, 5 min, 6 min, 7 min, 8 min, 9 min, 10 min), 2 mL of the reacted solution was filtered through a 0.22 μm membrane to remove the photocatalyst. The degradation of ABDA was monitored by measuring absorbance changes at a specific wavelength of 380 nm using a UV-Vis spectrophotometer, thereby detecting the generation of ^1^O_2_ during the photocatalytic reaction.

**1.7 Hydrogen Peroxide Decomposition Test**

Disperse 3 mg of COF photocatalyst in 45 mL of a 1 mM H_2_O_2_ aqueous solution. Prior to illumination, purge the system with nitrogen for half an hour to remove oxygen from the air that could participate in the reaction. Continue purging with nitrogen throughout the reaction to prevent the hydrogen peroxide generated from interfering with the decomposition experiment. The reaction duration was 2 h. Every half hour, 1 mL of the reaction solution was filtered through a 0.22 μm filter to remove the photocatalyst, and this sample was used to measure the hydrogen peroxide concentration at each time point. Changes in hydrogen peroxide concentration before and after illumination were determined using a UV spectrophotometer and the iodometric method.

**1.8 Rotating Ring-Disc Electrode (RRDE) Measurement**

The RRDE-3A Rotating Ring-Disk Electrode Assembly (Ver. 2.0) was used as the working electrode substrate. An RRDE equipped with a glassy carbon disk (area: 0.248 cm^2^) and a platinum ring (area: 0.187 cm^2^) was employed as the working electrode. The preparation method was the same as that used for RDE testing. Voltammetric curves were recorded in 0.1 M PBS solution at room temperature under an Ar or O_2_ protective atmosphere, with a scan rate of 10 mV s^-1^ and a rotation speed of 1000 rpm. The ring electrode potentials were set to 0.23 V and 0.6 V (vs. Ag/AgCl), respectively, for the detection of O_2_ and H_2_O_2_.

**1.9 Calculation of Apparent Quantum Yield (AQY)**

To evaluate the apparent quantum yield (AQY), hydrogen peroxide (H_2_O_2_) generation experiments were conducted under illumination through bandpass glass filters at 460 nm, 520 nm, 578 nm, and 630 nm. The light intensity at these wavelengths was measured using a power meter. The AQY was then calculated using the following formula:

**𝐴𝑄𝑌 (%) = 𝑁_ℎ_ / 𝑁_𝑝_ × 100% = 2 × 𝑁_𝐴_ × 𝑀 × ℎ × 𝑐 / 𝑃 × 𝑆 × 𝜆 × 𝑡 × 100%**

Here, n_p_ is the number of moles of the product (mol), N_A_ is Avogadro's number (6.022 × 10^23^ mol^-1^), P is the light intensity (W m^-2^), S is the illuminated area (m^2^), t is the duration of illumination (s), λ is the wavelength of the monochromatic light (m), and h is Planck's constant (6.626 × 10^-34^ J s), and c is the speed of light (3 × 10^8^ m s^-1^)。

**1.10 Electron Paramagnetic Resonance (EPR) Measurements**

Spin-capture EPR measurements were performed using a Bruker ELEXSYS II E 500 EPR spectrometer. 5,5-Dimethyl-1-pyrrolidine N-oxide (DMPO) was used as the spin trap to detect ‧OH and ‧O_2_^-^, tetramethylpiperidine oxide (TEMPO) was used to detect h^+^, and 2,2,6,6-tetramethylpiperidine (TEMP) was used to detect ^1^O_2_. The specific procedure is as follows: Disperse the catalyst (2 mg) in water or a methanol/water mixture (9:1, v/v, 500 μL), add DMPO, TEMPO or TEMP (0.1 mmol), and place the mixture in a Pyrex glass tube. Using a xenon lamp (λ = 467 nm) as the light source, bubble nitrogen or oxygen through the sample for 5–10 min prior to illumination. DMPO or TEMP spin-trap EPR spectra were acquired at 298 K, with signals recorded both in the dark and after 15 min of illumination.

**1.11 Calculation Details**

Geometry optimisations were performed using Gaussian 16^[1]^, employing the B3LYP functional ^[2]^ and 6-31G(d) ^[3]^ basis set. The geometry optimisations were run with dispersion corrections from Grimme’s D3 model ^[4]^ with Becke-Johnson damping factors ^[5]^. Harmonic vibrational frequencies calculations were performed to confirm the stationary points as true minima. Single-point energies are calculated using the 6- 311G (d,p) basis set.

For the analysis of molecular frontier orbital, molecular surface electrostatic potential, LOL-pi analysis of molecules, excited state electron and hole analysis, all of which are extracted by Multiwfn wave function analysis software ^[6]^ and analyzed and plotted by VMD software ^[7]^. TD-DFT method and CAM-B3LYP method are used to calculate the excited states of molecules. The adsorption energy is calculated by subtracting the energy of adsorbent and the energy of adsorbate from the energy of adsorption complex. The ball and stick structures were created using GaussView 6.1.1 ^[8]^.

**Part II: General Synthesis Methods**

**2.1 Synthesis of Monomers ^[9]^**

**Synthesis of Z-1**: To a 100-mL three-neck flask, add 2,4-dibromothiophene (93%) (2.601 g, 10 mmol), 8 mL toluene, and 8 mL of an aqueous solution of Na_2_CO_3_ (2.120 g, 20 mmol). Degas the mixture by bubbling nitrogen through it for 10 minutes. Dissolve 4-formylphenylboronic acid (1.499 g, 10 mmol) in 4 mL of EtOH and add it to the first solution. Further degas the mixture with bubbling N_2_ for 5 min. Then add Pd(PPh_3_)_4_ (0.165 g, 0.15 mmol) and reflux the mixture for 24 h. After the reaction, cool to room temperature, remove the solvent by evaporation, and add 50 mL of water. The mixture was then extracted three times with dichloromethane (DCM); the organic phases were combined, dried over MgSO_4_, and purified by silica gel column chromatography (elution: petroleum ether: DCM = 3:1) to afford the product (1.92 g), a white solid. The yield was 50%.^1^H NMR (400 MHz, 298 K, CDCl_3_, TMS): δ = 10.01 (s, 1H), 7.90 (d, *J* = 8.3 Hz, 2H), 7.71 (d, *J* = 8.3 Hz, 2H), 7.35 (s, 1H), 7.29 (s, 1H)。^13^C NMR (100 MHz, 298 K, CDCl_3_, TMS): δ = 192.32, 144.36, 138.69, 136.08, 130.54, 127.51, 125.94, 124.32, 111.21.

Figure S4. ^1^H NMR spectrum of Z-1 (400 MHz, 298 K, CDCl_3_, TMS).

**Figure S5.** ^13^C NMR spectrum of compound Z-1 (100 MHz, 298 K, CDCl_3_, TMS).

**Synthesis of Z-2**: In a 50 mL two-neck flask, combine compound Z-1 (1.069 g, 4 mmol), pinacol borate (610 mg, 2.4 mmol), Pd(dppf)Cl_2_ (29.3 mg, 0.4 mmol), and K_2_CO_3_ (2.211 g, 16 mmol). Degas the flask and purge with nitrogen three times, then add 15 mL of DMF. The mixture was degassed by bubbling nitrogen for 30 min and heated to 100 °C for 24 h. The solvent was then removed by rotary evaporation, 50 mL of water was added, and the mixture was extracted with chloroform. The organic phase was dried over MgSO_4_ and further purified by silica gel column chromatography (elution solvent: DCM: EA = 60:1), yielding the product as a yellow solid. The yield was 50%.^1^H NMR (400 MHz, 298 K, CDCl_3_, TMS): δ = 10.03 (s, 2H), 7.92 (s, 4H), 7.82 (d, *J* = 8.0 Hz, 4H), 7.70 (s, 2H), 7.49 (s, 2H)。^13^C NMR (100 MHz, 298 K, CDCl_3_, TMS): δ = 192.40, 142.82, 110.49, 138.29, 135.08, 130.95, 126.58, 125.25, 122.44.

Figure S6. ^1^H NMR spectrum of Z-2 (400 MHz, 298 K, CDCl_3_, TMS).

**Figure S7.** ^13^C NMR spectrum of compound Z-2 (100 MHz, 298 K, CDCl_3_, TMS)

**Synthesis of COThP-CHO**: In a 10-mL Pyrex tube, combine compound Z-2 (187 mg, 0.5 mmol), Pd(OAc)_2_ (17 mg, 0.075 mmol), Ag_2_CO_3_ (137.9 mg, 0.5 mmol), C_6_H_5_COOH (31 mg, 0.25 mmol), and toluene (4.0 mL). The Pyrex tube was cooled with liquid nitrogen, and the mixture was degassed three times via freeze-vacuum-freeze cycles. The mixture was stirred at 120°C for 24 h, then treated with an aqueous NaHCO_3_ solution and extracted with chloroform. The combined organic layers were dried over anhydrous Na_2_SO_4_ and evaporated to dryness. Further purification was performed by silica gel column chromatography (elution solvent: DCM: EA = 70:1), yielding a brown solid as the product. Yield: 43%. ^1^H NMR (400 MHz, 298 K, CDCl_3_, TMS): δ = 10.02 (s, 4H), 8.00 – 7.86 (m, 8H), 7.77 (d, *J* = 7.9 Hz, 8H), 7.47 (s, 4H)。^13^C NMR (100 MHz, 298 K, CDCl_3_, TMS): δ = 191.77，144.92，138.66，137.33，135.71，133.18，130.16，127.46，124.47。MS (MALDI-TOF): 744.0 [M]^+^; HR-MS (MALDI): *m/z* calcd. for C_44_H_24_O_4_S_4_ [M]^+1^: 744.0552, found: 743.9226.

Figure S8. ^1^H NMR spectrum of COThP-CHO (400 MHz, 298 K, CDCl_3_, TMS).

**Figure S9.** ^13^C NMR spectrum of compound COThP-CHO (100 MHz, 298 K, CDCl_3_, TMS).

**Figure S10.** MALDI-TOF Mass spectrum of COThP-CHO.

**2.2 Synthesis of NQ-COF_BD_ and NQ-COF_BD_-O**

Place COThP-CHO (11.17 mg, 0.015 mmol), biphenylamine (BD, 5.82 mg, 0.030 mmol), and ammonium iodide (17.4 mg, 0.12 mmol) into a Pyrex tube, then add tert-butyl peroxide (23 μL, 0.12 mmol), triethylamine (34 μL, 0.18 mmol), 1,4-dioxane (0.5 mL), mesitylene (0.5 mL), and an aqueous solution of 6 M acetic acid (0.1 mL). Sonicate the mixture for 5 min to obtain a uniformly dispersed solution. The Pyrex tube was cooled in liquid nitrogen, and air was removed from the tube by cycling through vacuum and nitrogen gas three times. The tube was then sealed under a nitrogen atmosphere, allowed to reach room temperature, and heated in an undisturbed oven at 130 °C for 72 h. After the oven temperature dropped to room temperature, the mixture was filtered to obtain a solid powder, which was washed several times with dichloromethane, 1,4-dioxane, and tetrahydrofuran. Finally, the purified product was placed in an oven at 120 °C and dried under vacuum for 12 h, yielding 13.0 mg of an orange, fluffy powder with a yield of 85.7%, designated as NQ-COF_BD_.

To synthesize NQ-COF_BD_-O, the corresponding NQ-COF_BD_ (49.3 mg, containing 0.05 mmol of quinoline linkers), *m*-CPBA (51.8 mg, 0.3 mmol total mass, 0.255 mmol active content), and DCM (3 mL) were added to a 10-mL Schlenk flask. The reaction mixture was then stirred for 1 hour at room temperature. The solid was isolated by vacuum filtration and washed successively with distilled water, tetrahydrofuran (THF), and DCM. Finally, after vacuum drying at 80 °C, 43.2 mg of a dark brown powder was obtained, yielding an 87.6% yield; this compound is designated as NQ-COF_BD_-O ^[10]^.

**2.3 Synthesis of NQ-COF_NA_ and NQ-COF_NA_-O**

Place COThP-CHO (11.17 mg, 0.015 mmol), naphthalene-2,6-diamine (NDA, 4.94 mg, 0.030 mmol), and ammonium iodide (17.4 mg, 0.12 mmol) into a Pyrex tube, followed by the sequential addition of tert-butyl peroxide (23 μL, 0.12 mmol), triethylamine (34 μL, 0.18 mmol), 1,4-dioxane (0.5 mL), mesitylene (0.5 mL), and an aqueous solution of 6 M acetic acid (0.1 mL). The mixture was sonicated for 5 min to obtain a uniformly dispersed solution. The Pyrex tube was cooled in liquid nitrogen, and air was removed from the tube by cycling through vacuum and nitrogen gas three times. The tube was sealed under a nitrogen atmosphere, allowed to reach room temperature, and then heated in an undisturbed oven at 130 °C for 72 h. After the oven temperature dropped to room temperature, the mixture was filtered to obtain a solid powder, which was washed several times with dichloromethane, 1,4-dioxane, and tetrahydrofuran. Finally, the purified product was placed in a 120 °C oven and dried under vacuum for 12 h, yielding 12.6 mg of brown, fluffy powder with a yield of 83.06%, designated as NQ-COF_NA_.

To synthesize NQ-COF_NA_-O, the corresponding NQ-COF_NA_ (49.3 mg, containing 0.05 mmol of quinoline linkers), *m*-CPBA (51.8 mg, 0.3 mmol total mass, 0.255 mmol active content), and DCM (3 mL) were added to a 10-mL Schlenk flask. The reaction mixture was then stirred for 1 hour at room temperature. The solid was isolated by vacuum filtration and washed successively with distilled water, tetrahydrofuran (THF), and DCM. Finally, after vacuum drying at 80 °C, 44.6 mg of a dark brown powder was obtained, yielding a 90.5% yield; this compound is designated as NQ-COF_NA_-O.

**Part III: Supplementary Charts and Tables**

**Figure S11.** SEM images of NQ-COF_BD_ (a, b) and NQ-COF_BD_-O (c, d).

**Figure S12.** High resolution S 2p XPS spectra of NQ-COF_BD_ (a) and NQ-COF_BD_-O (b).

**Figure S13.** XPS survey spectra of NQ-COF_BD_ (a) and NQ-COF_BD_-O (b).

**Figure S14.** The solid state ^13^C cross polarization magic angle spinning (^13^C CP/MAS) NMR spectrum of NQ-COF_BD_ (a) and NQ-COF_BD_-O (b).

**Figure S15.** EDS images of NQ-COF_BD_.

**Figure S16.** EDS images of NQ-COF_BD_-O.

**Table S1.** Elemental analysis result of COFs.

|  | **C %** | | **H %** | | **N %** | | **S %** | | **O %** | |
| --- | --- | --- | --- | --- | --- | --- | --- | --- | --- | --- |
|  | Exp. | Theor. | Exp. | Theor. | Exp. | Theor. | Exp. | Theor. | Exp. | Theor. |
| **NQ-COF_BD_** | 74.65 | 79.97 | 4.37 | 3.89 | 5.52 | 4.91 | 11.27 | 11.23 | 3.06 | 0 |
| **NQ-COF_BD_-O** | 68.59 | 75.73 | 3.60 | 3.68 | 4.25 | 4.65 | 9.76 | 10.64 | 7.63 | 5.31 |
| **NQ-COF_NA_** | 74.55 | 78.42 | 3.87 | 4.63 | 5.39 | 5.15 | 12.60 | 11.79 | 3.13 | 0 |
| **NQ-COF_NA_-O** | 66.99 | 74.06 | 3.37 | 4.38 | 4.49 | 4.87 | 9.83 | 11.14 | 7.72 | 5.56 |

**Figure S17.** Zeta-potentials of COFs.

**Table S2.** Structural Parameters and Refinement Results for COFs.

|  | NQ-COF_BD_ | NQ-COF_BD_-O |
| --- | --- | --- |
| a | 4.17652 Å | 4.58201 Å |
| b | 38.91643 Å | 39.43227 Å |
| c | 37.37687 Å | 34.56072 Å |
| α | 90° | 90° |
| β | 90° | 90° |
| γ | 90° | 90° |
| R_wp_ | 6.12% | 3.79% |
| R_p_ | 4.32% | 2.99% |

**Figure S18.** Pore width distribution profiles of NQ-COF_BD_ and NQ-COF_BD_-O.

**Figure S19.** Thermogravimetric analysis (TGA) curve of NQ-COF_BD_-O measured under nitrogen flow with a heating rate 10 ºC /min up to 800 ºC.

**Figure S20.** FT-IR spectra comparison of NQ-COF_BD_-O after immersing in different solvents for 3 days.

**Figure S21.** PXRD patterns of NQ-COF_BD_-O after being immersed in different organic solvents or acid/ base solutions for 3 days.

**Figure S22.** The Tauc plots of the NQ-COF_BD_ and NQ-COF_BD_-O for band gap calculation.

**Figure S23.** Mott-Schottky plot of NQ-COF_BD_ (a) and NQ-COF_BD_-O (b).

**Figure S24.** Water contact angles of NQ-COF_BD,_ NQ-COF_BD_-O, NQ-COF_NA_ and NQ-COF_NA_-O (all four COFs were pressed into thin slices for testing at room temperature in air).

**Figure S25.** PL analyses. Steady-state photoluminescence (PL) spectra of the NQ-COF_BD_, NQ-COF_BD_-O.

**Figure S26.** The corresponding electrostatic potential surface (ESP) maps of the NQ-COF_BD_ and NQ-COF_BD_-O models (The arrow in the figure indicates the direction of the dipole moment).

**Figure S27.** The photograph of the experimental setup under xenon lamp illumination, with the following illumination parameters: 300 W xenon lamp (λ ≥ 420 nm), irradiance of 100 mW·cm^-2^, illuminated area of 16π cm^2^, and reactor volume of 100 mL.

**Figure S28.** The Comparison of H_2_O_2_ Yields Catalyzed by NQ-COF_BD_-O at Different Solid-to-Liquid Ratios.

**Figure S29.** AQYs of NQ-COF_BD_ at different selected wavelengths (460, 520, 578, 630 nm).

**Figure S30.** H_2_O_2_ yield of NQ-COF_BD_-O under different pH reaction conditions.

**Figure S31.** Photocatalytic decomposition of H_2_O_2_ (C_0_ = 1 mM) in water under visible-light irradiation over COFs.

**Figure S32.** The formation (K_f_) and decomposition (K_d_) rate constants of H_2_O_2_ for COFs.

**Figure S33.** The continuous photocatalytic performance of the NQ-COF_BD_-O under Xenon lamp irradiation for consecutive 12 h.

**Figure S34.** FT-IR spectra of the NQ-COF_BD_-O before reaction(red) and after 12 h of photocatalytic reactions (black).

**Figure S35.** PXRD patterns of NQ-COF_BD_-O before reaction(red) and after 12 h of photocatalytic reactions (black).

**Figure S36.** SEM images of the NQ-COF_BD_-O before reaction (Left) and after 12 h of photocatalytic reactions (Right).

**Figure S37.** (a) High-resolution N 1s XPS spectrum of the fresh NQ-COF_BD_-O sample before the photocatalytic reaction. (b) High-resolution O 1s XPS spectrum of the fresh NQ-COF_BD_-O sample before the photocatalytic reaction. (c) XPS survey spectrum of the fresh NQ-COF_BD_-O sample before the photocatalytic reaction. (d) High-resolution N 1s XPS spectrum of NQ-COF_BD_-O after 12 h of photocatalytic reaction. (e) High-resolution O 1s XPS spectrum of NQ-COF_BD_-O after 12 h of photocatalytic reaction. (f) XPS survey spectrum of NQ-COF_BD_-O after 12 h of photocatalytic reaction.

**Figure S38.** (a) EPR signals of the reaction solution in the dark in the presence of DMPO as the spin-trapping reagent of NQ-COF_BD_ and NQ-COF_BD_-O. (b) EPR signals of the reaction solution in the dark in the presence of TEMP as the spin-trapping reagent of NQ-COF_BD_ and NQ-COF_BD_-O.

**Figure S39.** (a) EPR spectra of the reaction solution using DMPO as the spin-trapping agent for detecting ∙OH radicals over NQ-COF_BD_ and NQ-COF_BD_-O under dark and light conditions. (b) EPR spectra recorded with TEMPO as the trapping agent for detecting h⁺ over NQ-COF_BD_ and NQ-COF_BD_-O. (c) Relative EPR signal intensity of TEMPO used as the probe for photogenerated holes (h⁺) over NQ-COF_BD_ and NQ-COF_BD_-O (Blank represents the signal without catalyst).

**Figure S40.** The NBT method was used to detect ‧O_2_^-^ in the NQ-COF_BD_ and NQ-COF_BD_-O photocatalytic system, and the rates of ‧O_2_^-^ generation were compared.

**Figure S41.** The ABDA method was used to detect ^1^O_2_ in the NQ-COF_BD_ and NQ-COF_BD_-O photocatalytic system.

**Figure S42.** RRDE voltammograms of NQ-COF_BD_-O with a potential of 0.60V vs. Ag/AgCl on Pt ring electrode to detect H_2_O_2_.

**Figure S43.** (a) RRDE voltammograms of NQ-COF_BD_ with a potential of −0.23V vs. Ag/AgCl on Pt ring electrode to detect O_2_. (b) RRDE voltammograms of NQ-COF_BD_ with a potential of 0.60V vs. Ag/AgCl on Pt ring electrode to detect H_2_O_2_.

**Figure S44.** The calculated O_2_ adsorption energy on different sites of the NQ-COF_BD_.

**Figure S45.** The calculated O_2_ adsorption energy on different sites of the NQ-COF_BD_-O.

**Figure S46.** The solid state ^13^C cross polarization magic angle spinning (^13^C CP/MAS) NMR spectrum of NQ-COF_NA_ (a) and. NQ-COF_NA_-O (b).

**Figure S47.** C 1s (a), N 1s (b), O 1s (c) and S 2p (d) XPS analyses of NQ-COF_NA_ and NQ-COF_NA_-O.

**Figure S48.** XPS survey spectra of NQ-COF_NA_ (a) and NQ-COF_NA_-O (b).

**Figure S49.** FT-IR spectra of NQ-COF_NA_ and NQ-COF_NA_-O.

**Figure S50.** EDS images of NQ-COF_NA_.

**Figure S51.** EDS images of NQ-COF_NA_-O.

**Figure S52.** Pawley refinements of NQ-COF_NA_ (a) and NQ-COF_NA_-O (b) against their experimental PXRD patterns.

**Table S3.** Structural Parameters and Refinement Results for COFs.

|  | NQ-COF_NA_ | NQ-COF_NA_-O |
| --- | --- | --- |
| a | 5.36217 Å | 4.55466 Å |
| b | 36.69830 Å | 36.87929 Å |
| c | 31.77030 Å | 29.95747 Å |
| α | 90° | 90° |
| β | 90° | 90° |
| γ | 90° | 90° |
| R_wp_ | 5.56% | 4.04% |
| R_p_ | 4.26% | 3.10% |

**Figure S53.** TEM images of NQ-COF_NA_ (left) and NQ-COF_NA_-O (right).

**Figure S54.** N_2_ sorption analyses (a) and Pore width distribution profiles (b) of NQ-COF_NA_ and NQ-COF_NA_-O.

**Figure S55.** SEM images of NQ-COF_NA_ (a, b) and NQ-COF_NA_-O (c, d).

**Figure S56.** Thermogravimetric analysis (TGA) curve of NQ-COF_NA_-O measured under nitrogen flow with a heating rate 10 ºC /min up to 800 ºC.

**Figure S57.** FT-IR spectra comparison of NQ-COF_NA_-O after immersing in different solvents for 3 days.

**Figure S58.** PXRD patterns of NQ-COF_NA_-O after being immersed in different organic solvents or acid/ base solutions for 3 days.

**Figure S59.** The UV-Vis DRS (a) and The Tauc plots (b) of the NQ-COF_NA_ and NQ-COF_NA_-O.

**Figure S60.** Mott-Schottky plot of NQ-COF_NA_ (a) and NQ-COF_NA_-O (b).

**Figure S61.** The experimentally derived energy band alignments of the NQ-COF_NA_ and NQ-COF_NA_-O.

**Figure S62.** AQYs of NQ-COF_NA_ at different selected wavelengths (460, 520, 578, 630 nm).

**Figure S63.** AQYs of NQ-COF_NA_-O at different selected wavelengths (460, 520, 578, 630 nm).

**Figure S64.** The continuous photocatalytic performance of the NQ-COF_NA_-O under Xenon lamp irradiation for consecutive 12 h.

**Figure S65.** The photocatalytic performances of NQ-COF_NA_-O in different kinds of water under Xenon lamp irradiation.

**Figure S66.** Recycling experiments involving the photocatalytic Production of H_2_O_2_ with NQ-COF_NA_-O as the photocatalyst.

**Figure S67.** FT-IR spectra of the NQ-COF_NA_-O before reaction (red) and after 12 h of photocatalytic reactions (black).

**Figure S68.** PXRD patterns of NQ-COF_NA_-O before reactions (red) and after 12 h of photocatalytic reactions.

**Figure S69.** SEM images of the NQ-COF_NA_-O before reactions (left) and after 12 h of photocatalytic reactions. reactions (right).

**Figure S70.** (a) High-resolution N 1s XPS spectrum of the fresh NQ-COF_NA_-O sample before the photocatalytic reaction. (b) High-resolution O 1s XPS spectrum of the fresh NQ-COF_NA_-O sample before the photocatalytic reaction. (c) XPS survey spectrum of the fresh NQ-COF_NA_-O sample before the photocatalytic reaction. (d) High-resolution N 1s XPS spectrum of NQ-COF_NA_-O after 12 h of photocatalytic reaction. (e) High-resolution O 1s XPS spectrum of NQ-COF_NA_-O after 12 h of photocatalytic reaction. (f) XPS survey spectrum of NQ-COF_NA_-O after 12 h of photocatalytic reaction.

**Figure S71.** Different gas atmospheres of NQ-COF_NA_-O.

**Figure S72.** The NBT method was used to detect ‧O_2_^-^ in the NQ-COF_NA_ and NQ-COF_NA_-O photocatalytic system, and the rates of ‧O_2_^-^ generation were compared.

**Figure S73.** The ABDA method was used to detect ^1^O_2_ in the NQ-COF_NA_ and NQ-COF_NA_-O photocatalytic system.

**Figure S74.** The HOMO and LUMO orbital distributions of the simplified NQ-COF_NA_ and NQ-COF_NA_-O segments based on DFT calculation.

**Figure S75.** The corresponding electrostatic potential surface (ESP) maps of the NQ-COF_NA_ and NQ-COF_NA_-O models (The arrow in the figure indicates the direction of the dipole moment).

**Figure S76.** Hole-electron distribution heat map of each fragment in the NQ-COF_NA_ and NQ-COF_NA_-O molecule in the first excited singlet state (S₁).

**Table 4** Summary of the H_2_O_2_ production performance over COF-based photocatalysts.

| Entry | COF | Reaction  condition | Irradiation (nm) | H_2_O_2_ yield (μmol·h^-1^g^-1^) | AQY | Ref. |
| --- | --- | --- | --- | --- | --- | --- |
| 1 | **NQ-COF_BD_-O** | **H_2_O** | **λ > 420 nm** | **4569** | **6.1%@460nm** | **This**  **Work** |
| 2 | **NQ-COF_BD_** | **H_2_O** | **λ > 420 nm** | **2416** | **4.6%@460nm** |  |
| 3 | **NQ-COF_NA_-O** | **H_2_O** | **λ > 420 nm** | **2803** | **9.9%@460nm** |  |
| 4 | **NQ-COF_NA_** | **H_2_O** | **λ > 420 nm** | **1508** | **2.9%@460nm** |  |
| 5 | **NQ-COF_BD_-O** | **H_2_O : IPA = (9 : 1)** | **λ > 420 nm** | **10189** | **––** |  |
| 6 | **NQ-COF_BD_** | **H_2_O : IPA = (9 : 1)** | **λ > 420 nm** | **7060** | **––** |  |
| 7 | **NQ-COF_NA_-O** | **H_2_O : IPA = (9 : 1)** | **λ > 420 nm** | **3565** | **––** |  |
| 8 | **NQ-COF_NA_** | **H_2_O : IPA = (9 : 1)** | **λ > 420 nm** | **3368** | **––** |  |
| 9 | HHT-COF | H_2_O | λ ≥ 420 nm | 6432 | 8.77%@420nm | 11 |
| 10 | THT-COF | H_2_O | λ ≥ 420 nm | 5455 | 6.50%@420nm | 11 |
| 11 | THB-COF | H_2_O | λ ≥ 420 nm | 1366 | 2.01%@420nm | 11 |
| 12 | TY-COF | H_2_O | λ > 420 nm | 6455 | 9.5%@450nm | 12 |
| 13 | TE-COF | H_2_O | λ > 420 nm | 4804 | 6.0%@450nm | 12 |
| 14 | PAQ-TBZ | H_2_O | Xe lamp | 1236 | –– | 13 |
| 15 | PAQ-TABPB | H_2_O | Xe lamp | 3351 | 6.36%@400nm | 13 |
| 16 | PAQ | H_2_O | Xe lamp | 324 | –– | 13 |
| 17 | N-COF | H_2_O | λ =380 nm | 491 | –– | 14 |
| 18 | S-COF | H_2_O | λ =380 nm | 759 | –– | 14 |
| 19 | N-COF* | H_2_O | λ =380 nm | 1142 | –– | 14 |
| 20 | S-COF* | H_2_O | λ =380 nm | 3324 | –– | 14 |
| 21 | iBPyP-33 | H_2_O | Xe lamp | 3070 | –– | 15 |
| 22 | BPhP | H_2_O | Xe lamp | 1615 | –– | 15 |
| 23 | iBPyP-34 | H_2_O | Xe lamp | 1576 | –– | 15 |
| 24 | iBPyP-43 | H_2_O | Xe lamp | 2196 | –– | 15 |
| 25 | Co-COF-C_4_N | H_2_O : IPA = (9 : 1) | λ ≥ 420 nm | 12328 | 8.8%@420nm | 16 |
| 26 | COF-C_4_N | H_2_O : IPA = (9 : 1) | λ ≥ 420 nm | 8893 | 5.7%@420nm | 16 |
| 27 | AZA-CMP | H_2_O | λ ≥ 420 nm | 2770 | –– | 16 |
| 28 | C_4_N-CMP | H_2_O | λ ≥ 420 nm | 3382 | –– | 16 |
| 29 | B[f]QCOFs-1 | H_2_O | λ ≥ 420nm | 9025 | 8.9%@450nm | 17 |
| 30 | B[f]QCOFs-2 | H_2_O | λ ≥ 420nm | 5302 | –– | 17 |
| 31 | B[f]QCOFs-3 | H_2_O | λ ≥ 420nm | 5712 | –– | 17 |
| 32 | B[f]QCOFs-4 | H_2_O | λ ≥ 420nm | 2540 | –– | 17 |
| 33 | Ace-asy-CTF | H_2_O | λ ≥ 420nm | 2534 | 4.4%@420nm | 18 |
| 34 | COF-IN-1 | H_2_O | λ > 420 nm | 5463 | –– | 19 |
| 35 | COF-IN-2 | H_2_O | λ > 420 nm | 6141 | –– | 19 |
| 36 | COF-OUT-1 | H_2_O | λ > 420 nm | 983 | –– | 19 |
| 37 | COF-OUT-2 | H_2_O | λ > 420 nm | 2351 | –– | 19 |
| 38 | Por-COF-cya | H_2_O | λ > 420nm | 2209 | 3.82%@420nm | 20 |
| 39 | TTF@ Por-COF-cya | H_2_O | λ > 420nm | 6994 | 14.98%@420nm | 20 |
| 40 | Por-COF-cya | H_2_O : BA = (9 : 1) | λ > 420nm | 8979 | 3.82%@420nm | 20 |
| 41 | TTF@ Por-COF-cya | H_2_O : BA = (9 : 1) | λ > 420nm | 3218 | 14.98%@420nm | 20 |
| 42 | TP-DBS-COF | H_2_O | λ > 420 nm | 1320 | –– | 21 |
| 43 | TPDBS-COF/ZSM-5-15% | H_2_O | λ > 420 nm | 1670 | –– | 21 |
| 44 | TPDBS-COF/ZSM-5-30% | H_2_O | λ > 420 nm | 1830 | –– | 21 |
| 45 | TPDBS-COF/ZSM-5-45% | H_2_O | λ > 420 nm | 2010 | –– | 21 |
| 46 | TPDBS-COF/ZSM-5-60% | H_2_O | λ > 420 nm | 1930 | –– | 21 |
| 47 | TPDBS-COF/ZSM-5-15% | H_2_O : BA = (9 : 1) | λ > 420 nm | 2960 | –– | 21 |
| 48 | COF-4 | H_2_O | λ ≥ 420nm | 1839 | –– | 22 |
| 49 | FS-OH-COF | H_2_O | λ>420 nm | 390 | –– | 23 |
| 50 | FS-OMe-COF | H_2_O | λ>420 nm | 575 | –– | 23 |
| 51 | FS-OHOMe-COF | H_2_O | λ>420 nm | 784 | 9.6%@420nm | 23 |
| 52 | TCMP-1 | H_2_O | λ > 400 nm | 783.9 | –– | 24 |
| 53 | TCMP-2 | H_2_O | λ > 400 nm | 339.5 | –– | 24 |
| 54 | TCMP-3 | H_2_O | λ > 400 nm | 407.2 | –– | 24 |
| 55 | PCMP-1 | H_2_O | λ > 400 nm | 130.9 | –– | 24 |
| 56 | PCMP-2 | H_2_O | λ > 400 nm | 107.0 | –– | 24 |
| 57 | PCMP-3 | H_2_O | λ > 400 nm | 103.4 | –– | 24 |
| 58 | BTZ-3 | H_2_O : BA = (9 : 1) | λ > 420 nm | 3267 | –– | 25 |
| 59 | BTZ-1 | H_2_O : BA = (9 : 1) | λ > 420 nm | 1140 | –– | 25 |
| 60 | BTZ-3 | H_2_O | λ > 420 nm | 650 | –– | 25 |
| 61 | Mo-DHTA | H_2_O : EtOH = (9 : 1) | λ = 467 nm | 4084 | –– | 26 |
| 62 | Mo-DHTA | H_2_O : BA = (9 : 1) | λ = 467 nm | 626 | –– | 26 |
| 63 | Mo-DHTA | H_2_O | λ = 467 nm | 383 | –– | 26 |
| 64 | TAPB-DHTA COF | H_2_O : EtOH = (9 : 1) | λ = 467 nm | 232 | 7%@467nm | 26 |
| 65 | Mo-NH_2_ | H_2_O : EtOH = (9 : 1) | λ = 467 nm | 282 | –– | 26 |
| 66 | XJU-1 | H_2_O : BA = (9 : 1) | 350 < λ < 780 | 34777 | 9.7%@380nm | 27 |
| 67 | XJU-2 | H_2_O : BA = (9 : 1) | 350 < λ < 780 | 11922 | 9.4%@380nm | 27 |
| 68 | XJU-1 | H_2_O | 350 < λ < 780 | 7453 | 9.7%@380nm | 27 |
| 69 | XJU-2 | H_2_O | 350 < λ < 780 | 8124 | 9.4%@380nm | 27 |
| 70 | 2-SO_3_H-COF | H_2_O : BA = (9 : 1) | λ > 420 nm | 11470 | 5.36%@420nm | 28 |
| 71 | 2-SO_3_H-COF | pH=1 | λ > 420 nm | 4744 | 5.36%@420nm | 28 |
| 72 | BP-COF | H_2_O : BA = (9 : 1) | λ > 420 nm | 5445 | 3.01%@420nm | 28 |
| 73 | BP-COF | H_2_O | λ > 420 nm | 669 | 3.01%@420nm | 28 |
| 74 | SO_3_H-COF | H_2_O | λ > 420 nm | 2714 | 4.67%@420nm | 28 |
| 75 | 2-SO_3_H-COF | H_2_O | λ > 420 nm | 2915 | 5.36%@420nm | 28 |
| 76 | C_4_N/MgAl-LDH | H_2_O : ETA | 300 W Xe lamp | 4747 | 3.26% | 29 |
| 77 | C_4_N/MgAl-LDH | H_2_O | 300 W Xe lamp | 2380 | 3.26% | 29 |
| 78 | LDH | H_2_O | 300 W Xe lamp | 140 | –– | 29 |
| 79 | C_4_ | H_2_O | 300 W Xe lamp | 410 | –– | 29 |
| 80 | C_4_/p-MgAl-LDH | H_2_O | 300 W Xe lamp | 831 | –– | 29 |
| 81 | COF-JLU-51 | H_2_O : IPA = (9 : 1) | 420 < λ < 780 | 4735 | 6.4%@420nm | 30 |
| 82 | COF-JLU-52 | H_2_O : IPA = (9 : 1) | 420 < λ< 780 | 2689 | –– | 30 |
| 83 | BTZ-3 | H_2_O : BA = (9 : 1) | λ > 420 nm | 3267 | –– | 31 |
| 84 | BTZ-1 | H_2_O : BA = (9 : 1) | λ > 420 nm | 1140 | –– | 31 |
| 85 | BTZ-3 | H_2_O | λ > 420 nm | 650 | –– | 31 |
| 86 | COF-P | H_2_O : EtOH = (9 : 1) | λ ≥ 420 nm | 270 | –– | 32 |
| 87 | COF-T | H_2_O : EtOH = (9 : 1) | λ ≥ 420 nm | 191 | 2.17%@420nm | 32 |
| 88 | COF-pytri | H_2_O : EtOH = (9 : 1) | λ ≥ 420 nm | 1046 | –– | 32 |

**References**

[1] Frisch, M. J. et al. Gaussian 16 Revision A.03 (Gaussian, Inc., Wallingford CT, 2016).
[2] Becke, Axel D. "Density‐functional thermochemistry. III. The role of exact exchange." The Journal of chemical physics 98.7 (1993): 5648-5652.
[3] Lee, Chengteh, Weitao Yang, and Robert G. Parr. "Development of the Colle-Salvetti correlation-energy formula into a functional of the electron density." Physical review B 37.2 (1988): 785.
[4] Grimme, Stefan, et al. "A consistent and accurate ab initio parametrization of density functional dispersion correction (DFT-D) for the 94 elements H-Pu." The Journal of chemical physics 132.15 (2010).
[5] Johnson, Erin R., and Axel D. Becke. "A post-Hartree-Fock model of intermolecular interactions: Inclusion of higher-order corrections." The Journal of chemical physics 124.17 (2006).
[6] Lu, Tian, and Feiwu Chen. "Multiwfn: A multifunctional wavefunction analyzer." Journal of computational chemistry 33.5 (2012): 580-592.
[7] Humphrey, William, Andrew Dalke, and Klaus Schulten. "VMD: visual molecular dynamics." Journal of molecular graphics 14.1 (1996): 33-38.
[8] GaussView, Version 6.1.1, Roy Dennington, Todd Keith, and John Millam, Semichem Inc., Shawnee Mission, KS, 2019.
[9] Wang, Shitao, et al. "A three-dimensional sp^2^ carbon-conjugated covalent organic framework." *Journal of the American Chemical Society* 143.38 (2021): 15562-15566.
[10] Pang, Huaji, et al. "Embedding Hydrogen Atom Transfer Moieties in Covalent Organic Frameworks for Efficient Photocatalytic C−H Functionalization." *Angewandte Chemie* 135.51 (2023): e202313520.
[11] Pan, Zi‐Xian, et al. "Sub‐Stoichiometric [6+ 6] Covalent Organic Frameworks for H_2_O_2_ Photogeneration in Air and Pure Water." *Advanced Functional Materials* (2025): e12475.
[12] Yue, Jie-Yu, et al. "Distinct oxygen reduction pathways for solar H_2_O_2_ production by regulating unsaturated bonds in covalent organic frameworks." *Chemical Science* 16.30 (2025): 13883-13892.
[13] Chi, Wenwen, et al. "Accelerated Exciton Dissociation and Charge Transfer via Third‐Motif Engineered Conjugated Polymers for Photocatalytic Circulation‐flow Synthesis of H_2_O_2_." *Angewandte Chemie International Edition* (2025): e202508690.
[14] Ma, Tian-Tian, et al. "Photochromic radical states in 3D covalent organic frameworks with zyg topology for enhanced photocatalysis." *National science review* 11.7 (2024): nwae177.
[15] Zhao, Wuzi, et al. "Coupling Ionic Polarization, Electron Sponge Effect by One‐Pot Hypercrosslinking, Pyridiniumnation of Photosensitizer‐Based Porous Organic Polymers for Enhancing Advanced Oxidation Process." *Advanced Functional Materials* (2025): 2425137.
[16] Lai, Yingchao, et al. "Hierarchical Regulation to Design and Construct High Efficient Phenazine‐Linked COFs for Photosynthesis H_2_O_2_ from Water and O_2_ or Air." *Advanced Functional Materials* (2025): e15942.
[17] Xie, Ke-Hui, et al. "Multicomponent one-pot construction of benzo [f] quinoline-linked covalent organic frameworks for H_2_O_2_ photosynthesis." *Nature Communications* 16.1 (2025): 3493.
[18] Zhang, Hao, et al. "Enhanced photocatalytic production of hydrogen peroxide by covalent triazine frameworks with stepwise electron transfer." *ACS Catalysis* 14.23 (2024): 17654-17663.
[19] Zhu, Ruo‐Meng, et al. "Inwardly directed linker propagation: constructing 3D covalent organic frameworks for efficient H_2_O_2_ photosynthesis." *Angewandte Chemie International Edition* 64.32 (2025): e202510293.
[20] Gong, Zihan, et al. "Porphyrin‐based vinylene‐linked 3D covalent organic framework with unprecedented cya topology for photocatalytic H_2_O_2_ production." *Angewandte Chemie* 137.13 (2025): e202423205.
[21] Feng, Jingyi, et al. "3D‐Printed COF/Zeolite Composites for Augmented Photocatalytic Hydrogen Peroxide Production." *Angewandte Chemie International Edition* (2025): e202508226.
[22] Liao, Yingling, et al. "Tuning Linkage and Dimensionality in Covalent Organic Frameworks for Enhanced Photocatalytic Hydrogen Peroxide Production." *ACS Catalysis* 15 (2025): 10144-10153.
[23] Shu, Chang, et al. "Mixed‐linker strategy for the construction of sulfone‐containing D–A–A covalent organic frameworks for efficient photocatalytic hydrogen peroxide production." *Angewandte Chemie International Edition* 63.22 (2024): e202403926.
[24] Wang, Pan, et al. "Triazine Engineering in Conjugated Microporous Polymers for Hydrogen Peroxide Photosynthesis." *Advanced Functional Materials* (2025): 2507907.
[25] Wang, Yuancheng, et al. "Facile Construction of Fully Conjugated Benzotristhiazole‐Based Covalent Organic Frameworks for Efficient Photocatalytic H_2_O_2_ Production." *Advanced Functional Materials* (2025): 2508550.
[26] Kumbhakar, Bidhan, et al. "A Dimolybdenum Paddlewheel Embedded Covalent Organic Framework for Photocatalytic Hydrogen Peroxide Generation." *Small* (2025): 2501823.
[27] Lan, Wei, et al. "Efficient Photocatalytic Synthesis of Hydrogen Peroxide Facilitated by Triptycene‐Based 3D Covalent Organic Frameworks." *Small* 21.17 (2025): 2501327.
[28] Yang, Kaicheng, et al. "Bisite Sulfonate‐Functionalized Covalent Organic Frameworks with Efficient and Dynamic Proton Transportation for Accelerating H_2_O_2_ Photosynthesis." *Advanced Functional Materials* (2025): e20900.
[29] Teng, Yuan, et al. "Interface‐Engineered C4N/MgAl‐LDH Heterostructure for High‐Performance Photocatalytic H_2_O_2_ Production." *Angewandte Chemie International Edition* 64.48 (2025): e202516296.
[30] Zhang, Zhenwei, et al. "Tris (triazolo) triazine‐Based Covalent Organic Frameworks for Efficiently Photocatalytic Hydrogen Peroxide Production." *Angewandte Chemie* 136.45 (2024): e202411546.
[31] Wang, Yuancheng, et al. "Facile Construction of Fully Conjugated Benzotristhiazole‐Based Covalent Organic Frameworks for Efficient Photocatalytic H_2_O_2_ Production." *Advanced Functional Materials* (2025): 2508550.
[32] Zheng, A. Yaxuan, et al. "Advancing solar-driven redox processes: pyrene-based covalent organic frameworks for efficient environmental remediation and H_2_O_2_ production." *Green Chemistry* 27.30 (2025): 9254-9267.
